# Genotype × Environment Interactions of Yield Traits in Backcross Introgression Lines Derived from *Oryza sativa* cv. Swarna/*Oryza nivara*

**DOI:** 10.3389/fpls.2016.01530

**Published:** 2016-10-19

**Authors:** Divya Balakrishnan, Desiraju Subrahmanyam, Jyothi Badri, Addanki Krishnam Raju, Yadavalli Venkateswara Rao, Kavitha Beerelli, Sukumar Mesapogu, Malathi Surapaneni, Revathi Ponnuswamy, G. Padmavathi, V. Ravindra Babu, Sarla Neelamraju

**Affiliations:** Crop Improvement Section, ICAR- National Professor Project, ICAR- Indian Institute of Rice ResearchHyderabad, India

**Keywords:** BILs, stability, AMMI, GGE, *Oryza nivara*, yield traits

## Abstract

Advanced backcross introgression lines (BILs) developed from crosses of *Oryza sativa* var. Swarna/*O. nivara* accessions were grown and evaluated for yield and related traits. Trials were conducted for consecutive three seasons in field conditions in a randomized complete block design with three replications. Data on yield traits under irrigated conditions were analyzed using the Additive Main Effect and Multiplicative Interaction (AMMI), Genotype and Genotype × Environment Interaction (GGE) and modified rank-sum statistic (*YSi*) for yield stability. BILs *viz.*, G3 (14S) and G6 (166S) showed yield stability across the seasons along with high mean yield performance. G3 is early in flowering with high yield and has good grain quality and medium height, hence could be recommended for most of the irrigated locations. G6 is a late duration genotype, with strong culm strength, high grain number and panicle weight. G6 has higher yield and stability than Swarna but has Swarna grain type. Among the varieties tested DRRDhan 40 and recurrent parent Swarna showed stability for yield traits across the seasons. The component traits thousand grain weight, panicle weight, panicle length, grain number and plant height explained highest genotypic percentage over environment and interaction factors and can be prioritized to dissect stable QTLs/ genes. These lines were genotyped using microsatellite markers covering the entire rice genome and also using a set of markers linked to previously reported yield QTLs. It was observed that wild derived lines with more than 70% of recurrent parent genome were stable and showed enhanced yield levels compared to genotypes with higher donor genome introgressions.

## Introduction

Improving rice production per unit area and per unit time will be a major challenge in future due to the expanding population of rice consumers in the world. The average yield of existing cultivars reached a plateau and now research is directed toward wild relatives of *Oryza* to explore novel genes that can improve yield traits. Wild relatives were widely explored as donors for stress resistance and less exploited for yield improvement because of non-preferable agronomic traits linked with them. Wild rice genotypes provide a diverse range of allelic variation due to their adaption to a wide range of environmental conditions. Wild and related genotypes are valuable resources to explore novel variations to widen the genetic background of cultivated rice (Brar and Khush, 1997; Tanksley and McCouch, 1997; Swamy and Sarla, 2008; Wickneswari et al., 2012). Introgression of chromosomal segments from wild species into cultivated species can also generate de-novo variations in the new genetic background (Wang et al., 2005).

Back cross introgression lines developed from wild and adapted genotypes are useful in diversifying existing germplasm in more usable form and also in discovering novel genes/QTLs. As BILs have maximum genome of recurrent parent with few donor segments, it is advantageous to use them for precise estimation of quantitative traits. Fixed BILs can be replicated and can be used to study their environment interactions. The evaluation of the BILs for stability is very important especially when it is derived from an interspecific cross, as it takes more time to attain stability in the new back ground. Utilization of stable BILs will accelerate varietal development due to the presence of novel genes in an adapted parental background (Jeuken and Lindhout, 2004).

As grain yield is a complex quantitative trait, with high environmental interaction; selection of genotypes based on performance in single environment is not effective for varietal identification (Shrestha et al., 2012). It is essential to carry out selection based on yield stability evaluation than average performance in multiple environment conditions (Kang, 1993; Tariku et al., 2013; Islam et al., 2015). Selection of genotypes for stability and adaptability is required prior to recommendation in case of a crop such as rice which is grown in diverse ecologies. Stability is the suitability of a variety over a wide range of environments while adaptability is the better survival of a genotype over any specific environment. This can be attained through either genetic or physiological homeostasis of genotypes for environmental fluctuations (Singh and Narayanan, 2006). For cultivation in large area it is stability for yield traits which is desirable but for achieving maximum productivity, it is adaptability to best target environments that is preferred.

Effects of genotype, environment and genotype × environment interaction determine the phenotypic performance and its general and specific adaptation to different environments (Falconer and Mackey, 1996). This information is required for planning better selection strategies and to identify the best environment to select genotypes for grain yield (Gauch and Zobel, 1996; Kang, 1998). Several studies have been conducted on stability performance for grain yield of rice for different ecosystems (Cooper et al., 1999; Wade et al., 1999; Ouk et al., 2007; Anandan et al., 2009; Kumar et al., 2012; Tariku et al., 2013; Liang et al., 2015; Katsura et al., 2016). Many such studies showed that genotype × environment interaction was more significant than genotypic main effects (Henderson et al., 1996; Cooper and Somrith, 1997; Wade et al., 1997, 1999; Cooper et al., 1999; Inthapanya et al., 2000).

There are several methods to study stability and genotype × environment interactions of traits through conventional analysis. Different models were proposed on stability variance, ecovalence, regression coefficient analysis or principal component analysis (PCA) (Finlay and Wilkinson, 1963; Eberhart and Russell, 1966; Perkins and Jinks, 1968; Freeman and Perkins, 1971; Shukla, 1972; Kang, 1993). Kang (1993) proposed yield stability static (Ysi) by combining yield and stability as a single selection criterion by modifying rank Sum method. However, additive main effects and multiplicative interaction (AMMI) model and the genotype main effects and genotype × environment interaction effects (GGE) model are more popular methods. This method is followed to quantify the genotype environment interaction through PCA and graphical representation and has been widely applied in the multi-environment cultivar trials (Kempton, 1984; Crossa et al., 1990; Gauch and Zobel, 1997).

A panel of 14 BILs derived from Swarna/ *Oryza nivara* was studied along with 9 high yielding rice varieties of different duration and these 23 lines were screened in three seasons. Genotypic characterization of these BILs was conducted with genome wide polymorphic markers and markers linked to yield QTLs. The objectives of this study were (1) to identify the yield potential of backcross introgression lines in comparison with existing popular varieties (2) to identify stable high yielding BILs and their parental genome percentage (3) to prioritize the component traits important for further genetic dissection and improvement.

## Materials and methods

### Location

Field experiments were conducted at Indian Institute of Rice Research, Hyderabad (17° 19′ N and 78° 29′ E) at an altitude of 549 m above mean sea level during two wet seasons *Kharif* -2013(E1), (*Kharif* -2014) (E2) and one dry season *Rabi*-2014(E3). Crop was grown in alkaline vertisol with a pH of 7.94 at irrigated field conditions. Details of meteorological conditions during the crop growth period are presented in Table 1.

**Table 1:** **Weather parameters during crop season**.

**Season**	**Months**	**Temperature (°C)**			**R.H. (%)**		**Rainfall (mm)**	**Rainy days**	**Sunshine (Hrs.)**	**Wind speed (Km/Hr)**	**Evaporation (mm)**	**Crop stage**
		**Max**.	**Min**.	**Mean temp**.	**I**	**II**						
*Kharif-2013*	Jul-13	32.64	23.92	28.25	81.83	56.83	150.20	9.00	4.56	10.55	4.69	Sowing
	Aug-13	28.49	21.94	25.21	89.90	75.93	158.10	1.00	3.39	6.36	4.02	Transplanting
	Sep-13	31.05	20.59	25.82	87.07	64.33	110.60	8.00	5.71	3.05	4.69	Vegetative stage
	Oct-13	29.99	19.73	24.86	88.77	63.33	253.20	9.00	5.45	2.87	3.86	Heading / observations on Pn
	Nov-13	28.42	14.38	21.40	86.27	50.43	31.00	2.00	6.66	1.72	2.69	Harvesting
	Mean	30.54	21.54	26.04	86.89	65.11	168.03	6.75	4.78	5.71	4.32	
*Rabi*-2014	Dec-13	28.02	10.09	19.05	83.10	36.58	0.00	0.00	8.87	1.75	2.70	Sowing
	Jan-14	28.69	13.25	20.97	84.74	40.29	0.00	0.00	8.17	2.49	3.07	Transplanting
	Feb-14	31.20	16.55	23.88	78.36	32.75	0.00	0.00	8.99	2.93	4.62	Vegetative stage
	Mar-14	33.23	20.36	26.80	79.58	36.39	56.80	5.00	7.35	2.69	4.61	Vegetative stage
	Apr-14	37.60	22.04	29.82	76.73	36.07	72.60	2.00	7.72	2.02	6.02	Heading / observations on Pn
	May-14	37.74	23.87	30.81	66.10	33.84	40.10	3.00	8.39	3.47	7.11	Heading / observations on Pn
	Jun-14	37.02	24.60	30.81	68.57	45.07	53.60	3.00	7.90	10.17	8.14	Harvesting
	Mean	33.69	19.22	26.45	77.10	35.87	44.62	2.00	8.13	2.72	5.09	
*Kharif-2014*	Jul-14	31.80	23.60	27.70	82.32	62.40	108.00	8.00	3.20	12.40	4.00	Sowing
	Aug-14	32.00	23.30	27.65	85.90	62.70	184.10	10.00	5.40	7.10	3.00	Transplanting
	Sep-14	30.30	22.40	26.35	87.43	60.80	60.60	7.00	5.10	5.20	3.00	Vegetative stage
	Oct-14	31.60	19.90	25.75	83.45	49.80	69.20	3.00	6.40	2.40	4.80	Heading / observations on Pn
	Nov-14	30.60	16.80	23.70	79.23	40.90	10.60	1.00	7.00	1.80	4.70	Harvesting
	Mean	31.26	21.20	26.23	83.67	55.32	86.50	5.80	5.42	5.78	3.90	

### Plant material

Studies were conducted at IIRR to develop wild introgression lines between *O. sativa cv.* Swarna and accessions of wild relative, *Oryza nivara* (Kaladhar et al., 2008; Swamy et al., 2011). The developed BILs were advanced to BC_2_F_6_ generation and further purified by single panicle selection method upto BC_2_F_8_. From two sets of BILs consisting of 94 lines from Swarna / *O. nivara* (81848) (S lines) and 104 lines from Swarna / *O. nivara* (81832) (K lines), a panel of 14 BILs at BC_2_F_8_ generation were selected based on their preferable phenotypic traits (Table 2).

**Table 2:** **Description of the genotypes used in the study**.

**Code**	**Genotype**	**Type**	**Pedigree**	**Year of release**	**Origin**
G1	14_3	BIL	Swarna / *O. nivara*	–	IIRR, India
G2	148S	BIL	Swarna / *O. nivara*	–	IIRR, India
G3	14S	BIL	Swarna / *O. nivara*	–	IIRR, India
G4	166_1S	BIL	Swarna / *O. nivara*	–	IIRR, India
G5	166_2S	BIL	Swarna / *O. nivara*	–	IIRR, India
G6	166S	BIL	Swarna / *O. nivara*	–	IIRR, India
G7	248S (DRR Dhan 40)	BIL	Swarna / *O. nivara*	2013	IIRR, India
G8	24K	BIL	Swarna / *O. nivara*	–	IIRR, India
G9	250K	BIL	Swarna / *O. nivara*	–	IIRR, India
G10	3_1K	BIL	Swarna / *O. nivara*	–	IIRR, India
G11	65S	BIL	Swarna / *O. nivara*	–	IIRR, India
G12	70S	BIL	Swarna / *O. nivara*	–	IIRR, India
G13	75S	BIL	Swarna / *O. nivara*	–	IIRR, India
G14	7K	BIL	Swarna / *O. nivara*	–	IIRR, India
G15	Swarna	*indica*	Vasistha/Mahsuri	1982	ARI, Maruteru
G16	IR64	*indica*	IR5657-33-2-1/ IR2061-465-1-5-5	1991	IRRI
G17	Jaya	*indica*	TN-1/T-141	1968	
G18	MTU1010	*indica*	Krishnaveni/IR64	2000	ARI, Maruteru
G19	MTU1081	*indica*	Ajaya/BPT5204		ARI, Maruteru
G20	NLR34449	*indica*			
G21	Sahbhagi Dhan	*indica*	IR55419-4^*^2/WayRarem	2009	CRRI and IRRI
G22	Tellahamsa	*indica*	HR12/TN1	1971	
G23	Tulasi	*indica*	Rasi/Finegora	1988	IIRR, India

As BILs have a range of flowering duration from 77 to 120 days popular varieties IR64, Jaya, MTU1010, MTU1081, NLR34449, Sahbhagi Dhan, Swarna, Tellahamsa, and Tulasi with different flowering duration were grown as checks under irrigated conditions. These BILs were evaluated for yield and related traits in irrigated conditions over a period of three seasons (2013–2014) along with checks. As there is considerable variation in the duration among the BILs, per day productivity was computed to compare genotypes with different duration.

### Field experimental details

Seeds were sown in nursery beds, and 25 days old seedlings were transplanted, with single seedling per hill in all the field trials. The planting density was 33.3 hills m^−2^, with 20 cm row spacing and 15 cm intra-row spacing with five rows of 21 plants each constituting a replication. Normal package of practice and fertilizer application was followed; weeds, insects, and diseases were controlled by using standard herbicides and pesticides as required to avoid yield loss. The experimental plots were arranged in a randomized complete block design with three replications each containing 105 plants. These same parameters were followed uniformly across the seasons.

### Phenotyping

These genotypes were screened for various yield contributing traits in all the seasons following Standard Evaluation System (IRRI, 2013). The observations on yield and morpho-agronomic traits were recorded from the field experiments.

### Statistical analysis

Analysis of variance was computed for individual environment, then a combined analysis of variance was performed, considering both environments and genotypes as fixed using PB tools (Version 1.4, http://bbi.irri.org/products) and R (R Core Team, 2012). Significance of all effects was tested against mean square of error. The performance of BILs was tested over three seasons and was assessed using stability models *viz*, (1) yield-stability statistic (*YSi*) (Kang, 1993), (2) Additive Main effects and Multiplicative Interaction (AMMI) (Gauch and Zobel, 1997), and (3) GGE Biplot or Site Regression model (Yan and Kang, 2003). These models were used to interpret and visualize the stability and GEI patterns. In the AMMI model, only the GEI term is absorbed in the multiplicative component, whereas in the GGE model, the main effects of genotypes (G) plus the GEI are absorbed into the multiplicative component. Yield-stability (*YSi*) statistic was developed by Kang (1993) to be used as a selection criterion when G × E interaction is significant. The stability-variance was determined following modified Shukla's (1972) method and genotypes with significant stability variance were considered unstable. The stability variance was integrated with yield to obtain the *YSi* statistic as outlined by Kang and Magari (1995). Simultaneous selection of high yielding and stable genotypes is possible through this method.

The AMMI model (Gauch, 1988) was used in analyzing the stability and interaction for yield traits. The AMMI model is a combination of analysis of variance (ANOVA) and principal component analysis (PCA). The G × E interaction was evaluated with the AMMI model by considering the first two principal components. ANOVA model was used to analyze the trait data with main effects of genotype and environment without the interaction, then, a principal component analysis was integrated using the standardized residuals. These residuals include the experimental error and the effect of the GEI. The analytical model can be written as

$$\begin{array}{l} Y_{ij}=\mu +\delta_{i}+\beta_{j}+\sum_{K=1}^{K}\lambda_{k}\delta_{ik}\beta_{jk}+\varepsilon_{ij} \end{array}$$

Where *Y*_*ij*._ is the mean yield of *i*^th^ genotype in *j*^th^ environment, μ is the overall mean, δ_*i*_ is the genotypic effect, β_*j*_ is the environment effect, λ_*k*_ is the singular value for PC axis k, δ*ik* is the genotype eigenvector value for PC axis n, β_*jk*_ is the environment eigenvector value for PC axis k and ε_*ij*_ is the residual error assumed to be normally and independently distributed (0, σ2/r), σ2 is the pooled error variance and r is the number of replicates.

GGE biplots display both G (genotype) and GE (genotype environment) variation (Kang, 1993) for genotype evaluation. The GGE biplot is based on the sites regression (SREG) linear bilinear model (Cornelius et al., 1996; Crossa and Cornelius, 1997; Crossa et al., 2002). The sites regression model as a multiplicative model in the bilinear terms shows the main effects of cultivars plus the cultivar × environment interaction (GGE) and the model is

$$\begin{array}{l} Y_{ij}=\mu +\delta_{i}+\beta_{j}+\sum_{K=1}^{K}\lambda_{k}\delta_{ik}\beta_{jk}+\varepsilon_{ij} \end{array}$$

The GGE biplot graphically represents G and GEI effect present in the multi-location trial data using environment centered data. GGE biplots were used to evaluate (1) mega environment analysis (which-won-where pattern), where genotypes can be recommended to specific mega environments. (2) Genotype evaluation, where stable specific genotypes can be recommended across all locations and (3) location evaluation, explains discriminative power of target locations for genotypes under study.

Sum of square percentage was computed as percentage of sum of squares of components of stability analysis of variance per total sum of squares to know the contribution of each component viz., genotype, environment and GEI. Correlation analysis was performed with Statistical Tool for Agricultural Research (STAR) using Pearson's correlation coefficient method. Significance levels are indicated as: ^*^P < 0.05, ^**^P < 0.01, ^***^P < 0.001.

### Genotyping

**Figure 1:**
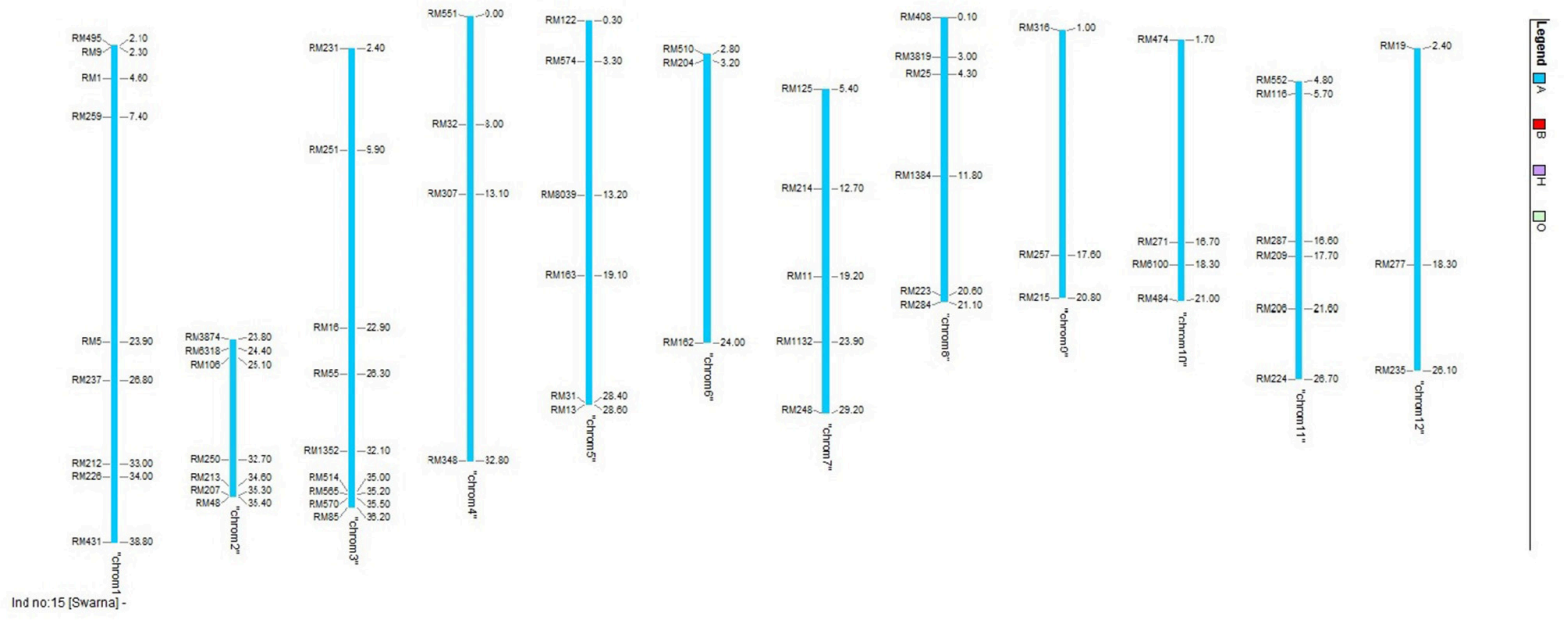
**Chromosomal location of the polymorphic markers used for genotyping in this study**.

Molecular screening was conducted to identify the presence of reported QTLs in the BILs and also to identify recurrent parent genome percentage. Leaves of 20 days-old seedlings were collected from the field and CTAB (Cetyl Trimethyl Ammonium Bromide) method was followed for DNA extraction (Doyle and Doyle, 1987). Polymorphic SSR markers with genome wide distribution (Figure 1) from universal core genetic map (Orjuela et al., 2010) were used for genotyping (Supplementary Table 1). PCR reactions were carried out in Thermal cycler (Veriti PCR, Applied Biosystems, USA) with the total reaction volume of 10 μl containing 15 ng of genomic DNA, 1X assay buffer, 200 μM of dNTPs, 1.5 mM MgCl_2_, 10 pmol of forward and reverse primer and 1 unit of Taq DNA polymerase (Thermo Scientific, U.S.A). PCR cycles were programmed as follows: initial denaturation at 94°C for 5 min followed by 35 cycles of 94°C for 45 s, 55°C for 30 s, 72°C for 45 s and a final extension of 10 min at 72°C. Amplified products were resolved in 4% metaphor agarose gels prepared in 0.5 X TAE buffer and electrophoresis was conducted at 120V for 2 h. Gels were stained with ethidium bromide and documented using gel documentation system (Alfa imager, U.S.A). Amplified fragments were scored for the presence (1) or absence (0) for each primer genotype combination. The SSR genotypic data generated in the population were analyzed using the software, GGT ver.2.0. The graphical representations and comparisons were made among the 23 lines on linkage group basis and also the entire genome level on individual basis.

## Results

### Yield and yield related traits

Wide range of variation was observed for yield traits among the genotypes and across the environments. Combined analysis of variance of three environmental data showed significant genotypic and genotype × environment interactions for all the traits except for 1000 grain weight where the G × E interactions were not significant. In the three environments *Kharif* 2013, *Rabi* 2014 and *Kharif* 2014 the variation in seasonal average was observed for DFF, GY, BY PH, TN, and BM. In three seasons; broad genotypic variation was observed and genotypic average ranged for DFF (77.37 to 133.04); GY (4.73 to 24.63); BY (0.33 to 2.10); PH (65.98 to 148.43); TN (6.12 to 20.70); GN (93.70 to 314.55); PL (18.09 to 25.19); PW (0.98 to 3.87); TGW (12.50 to 26.07); SF (60.57 to 97.57); BM (11.26 to 54.12); HI (0.20 to 0.55) and per day productivity (0.04 to 0.19) among the BILs under study (Table 3). The data obtained from the three replications was assessed and compared with high yielding checks in each season. Considering the three season average, among BILs G3 scored highest grain yield, harvest index and per day productivity and G6 scored highest bulk yield compared to Swarna and on par with other checks. G2 was of shortest duration and showed desirable yield traits such as panicle length, 1000 grain weight, spikelet fertility and plant height compared with checks. It was early in flowering with lowest unfilled grains in all the seasons. Derived lines from G6 i.e., G5 had highest grain number, filled grains and panicle weight. G8 was identified as having highest average biomass and dry matter production among BILs, G13, and G1 for high tiller number and G14 for maximum days to maturity. High yielding check MTU1010 showed highest grain yield, bulk yield, harvest index and per day productivity than BILs for three seasons average.

**Table 3:** **The mean performance of genotypes under the study across the seasons**.

**SL. NO**	**GENOTYPE**	**DFF**	**GY**	**BY**	**PH**	**TN**	**GN**	**PL**	**PW**	**TGW**	**SF**	**TDM**	**HI**	**YPD**
G1	14_3	108.67	6.18	0.43	74.66	13.89	122.96	21.58	1.25	13.72	66.13	19.04	0.33	0.05
G2	148S	92.00	15.21	1.18	139.73	11.40	112.56	24.28	2.64	24.99	93.60	39.73	0.38	0.13
G3	14S	103.67	20.87	1.56	82.58	12.20	111.06	22.04	2.25	23.49	89.28	37.09	0.54	0.15
G4	166_1	112.33	11.00	1.39	95.84	9.62	169.42	22.23	2.63	18.39	79.70	34.84	0.31	0.08
G5	166_2	108.33	15.11	1.21	87.89	8.20	261.76	21.35	3.65	18.18	63.53	37.85	0.39	0.11
G6	166S	112.44	18.90	1.67	94.62	10.38	183.97	22.07	3.16	19.63	81.99	41.78	0.44	0.13
G7	248S	117.00	14.61	1.42	83.83	12.01	123.22	20.25	1.96	16.48	71.27	37.64	0.38	0.10
G8	24K	119.89	16.58	1.35	106.67	9.44	154.33	19.72	2.73	18.67	83.11	50.53	0.35	0.11
G9	250K	117.67	13.02	0.94	92.60	11.96	128.21	22.04	2.08	17.89	81.59	36.04	0.35	0.09
G10	3_1K	115.78	12.26	1.19	83.51	13.22	140.03	19.63	1.85	16.62	76.47	34.47	0.35	0.08
G11	65S	111.22	12.96	1.14	111.69	13.56	149.81	20.44	1.92	14.18	83.57	38.75	0.33	0.09
G12	70S	116.33	12.63	1.26	76.34	13.68	127.18	18.75	1.77	17.51	89.00	35.97	0.34	0.08
G13	75S	114.67	13.31	1.11	72.42	14.38	112.17	19.25	1.90	17.04	86.21	29.65	0.44	0.09
G14	7K	122.11	16.67	1.11	90.59	12.81	162.06	22.53	2.12	16.21	72.84	47.04	0.36	0.11
G15	Swarna	120.72	18.13	1.36	86.56	13.82	156.99	20.84	2.10	16.91	75.57	45.86	0.41	0.13
G16	IR64	104.11	17.71	1.50	86.53	13.53	130.00	23.17	2.37	24.30	83.72	38.52	0.50	0.15
G17	JAYA	112.78	16.94	1.62	98.22	11.27	152.03	22.42	2.56	23.09	77.16	42.30	0.45	0.13
G18	MTU1010	97.11	21.19	1.86	95.53	11.40	144.44	22.39	2.47	24.28	78.81	39.84	0.54	0.17
G19	MTU1081	102.11	19.09	1.28	95.24	9.98	230.55	23.56	3.28	15.70	81.06	38.13	0.50	0.14
G20	NLR34449	104.56	14.72	1.38	76.64	13.84	154.89	19.24	1.72	13.42	77.51	31.49	0.47	0.11
G21	Sahbhagi Dhan	97.11	15.96	1.49	97.02	9.89	155.56	23.12	2.70	21.46	83.60	33.82	0.47	0.13
G22	Tellahamsa	93.11	15.46	1.37	100.38	11.60	137.69	23.28	2.75	23.14	84.22	34.99	0.44	0.12
G23	Tulasi	95.11	17.77	1.59	91.20	12.67	137.80	20.47	2.44	23.27	79.01	36.41	0.49	0.14
	Mean	108.64	15.49	1.32	92.19	11.95	150.38	21.51	2.36	19.07	79.95	37.47	0.42	0.11
	Max	122.11	21.19	1.86	139.73	14.38	261.76	24.28	3.65	24.99	93.60	50.53	0.54	0.17
	Min	92.00	6.18	0.43	72.42	8.20	111.06	18.75	1.25	13.42	63.53	19.04	0.31	0.05
	Variance	85.23	11.47	0.08	205.96	2.97	1291.03	2.42	0.31	13.46	50.24	39.37	0.00	0.00
	SD	9.23	3.39	0.29	14.35	1.72	35.93	1.56	0.56	3.67	7.09	6.27	0.07	0.03

The BILs were screened for seedling vigor in two seasons *Kharif* and *Rabi* in field conditions. Seedling vigor for BILs was obtained both for *Kharif* and *Rabi* season in terms of plant height and tiller number from the data taken on 40 days after transplanting and 70 days after transplanting. BILs seeds were subjected to germination test and vigor index analysis *in vitro* by paper towel method (ISTA, 1999). Seedling vigor was also assessed based on paper towel method using the data for shoot length and root length from 7 days and 14 days and germination studies. In both the seasons G2 had highest seedling vigor in terms of plant height. G14 was best for number of tillers in *Kharif* and G7 in *Rabi* season. G13(75S) showed highest vigor in terms of tiller number consistently across the seasons. In terms of vigor, G6 and derived lines were better compared to checks. Productive tillers were highest in G1 at the time of harvest. Among the checks Tulasi and Sahbhagi Dhan showed comparatively higher vigor and BILs outperformed popular checks. G2 showed highest seedling vigor in paper towel screening method.

### Stability analysis

Observations on the yield traits for all three seasons were then subjected to combined analyses through Yield-stability statistic (*YSi*) (Kang, 1993), AMMI and GGE biplot models. In the analysis, each combination of season with location was considered as an environment. Analysis of variance was first conducted for each environment. Pooled data of 3 seasons was subjected to stability analysis using PB tools and R software and specific genotypic adaptation, general genotypic adaptation and specific population adaptation to different seasons were identified.

It was found that G3 was the most stable genotype in the selection ranks for GY followed by G6, Swarna, G5, G7, and G14 based on combined analysis of yield and stability using *YSi* statistic. Similarly for bulk yield, G6 scored highest rank followed by G4, G7, G12, G3, G10, and G2. Among the BILs G6 and G3 showed non-significant stability variance and high average yield, so they may be considered for further multilocation trials. Number of genotypes selected based on *YSi* ranking varied among the traits. 9 genotypes were found to be superior based on *YSi* scoring for GY and PH; 8 for DTM, FG,TGW, BM, TDMPD;7 for BY, DFF, TN,PTN, GN,PL,SF, and YPD and 5 genotypes were selected based on high trait mean and stability for HI. G6 was found to be stable for 14 traits under study except DFF, TN, PTN, and BM followed by Swarna which was stable for 13 yield contributing traits. G14(7K) showed stability and high mean for 12 traits, G3(14s) for 11 traits, G2(148S) and G8(24K) for 10 traits and G7(248S) for 9 traits (Table 4).

**Table 4:** *****YSi*** Ranking of each genotype based on trait means and significance of stability variance**.

**SL. NO**	**YSi**	**GY**	**BY**	**DFF**	**DTM**	**PH**	**TN**	**PTN**	**GN**	**FG**	**PL**	**PW**	**TGW**	**SF**	**BM**	**TDM**	**HI**	**TDMPD**	**YPD**	**Rank sum**	***Ysi* Sum**
G1	14_3	−2	−2	1	−8	−1	15+	12+	−3	−10	2	−10	−2	−9	−2	−2	0	−2	−2	2	−25
G2	148S	3	5+	−10	−10	18+	4	4	0	−3	18+	6+	10+	18+	12+	12+	6	16+	10+	10	119
G3	14S	18+	8+	−9	−9	1	9+	11+	−10	2	4	9+	17+	17+	1	6+	18+	11+	18+	11	122
G4	166_1	−8	13+	−3	2	10+	1	0	7+	7+	15+	5+	12+	0	11+	3	−9	3	−8	8	61
G5	166_2	10+	−1	0	2	6	−1	−1	10+	10+	9+	14+	11+	−2	−4	6+	12+	5	10+	9	96
G6	166S	16+	18+	−2	5+	12+	3	3	9+	9+	14+	9+	16+	10+	5	13+	16+	12+	16+	14	184
G7	248S	9+	11+	14+	−6	3	6	5	−6	−9	3	4+	1	−8	7+	7+	11+	8+	9+	9	69
G8	24K	4	3	12+	9+	16+	0	2	3	6+	−6	8+	13+	11+	10+	10+	5	10+	4	10	120
G9	250K	−3	−8	15+	7+	11+	5	9+	5+	4+	12+	2	8+	9+	−2	−3	6	−3	−4	9	70
G10	3_1K	2	6+	11+	8+	−2	11+	11+	3	−2	1	−7	3	−3	3	2	4	2	1	5	54
G11	65S	4	4	−4	−2	17+	8+	13+	10+	12+	−3	3	−1	4	13+	11+	1	6+	2	8	98
G12	70S	−1	10+	4+	8+	−4	5	7+	−4	−1	−2	−9	7+	16+	10+	0	2	0	−2	7	46
G13	75S	−2	2	1	5+	−2	9+	6	−1	1	−1	2	5	14+	−5	−8	15+	−8	−3	4	30
G14	7K	9+	−5	10+	18+	0	10+	6	14+	11+	8+	8+	0	2	9+	8+	−1	6+	7+	12	120
G15	Swarna	15+	4	9+	14+	5	148	13+	8+	10+	6+	7+	4	−4	15+	15+	5	15+	15+	13	170
Trait Mean		14.50	1.22	112.86	142.61	91.97	12.04	11.32	147.71	115.93	21.13	2.27	17.99	79.59	23.25	37.75	0.38	0.26	0.10		
Ysi Mean		4.93	4.53	3.27	2.87	6.00	6.60	6.73	3.00	3.13	5.33	3.40	6.93	5.00	5.53	5.33	6.07	5.40	4.87		
L.S.D. (0.05)		2.45	0.19	1.93	1.71	3.57	2.14	2.09	11.47	11.70	0.83	0.23	0.74	4.09	3.36	5.05	0.04	0.04	0.15		

+ indicates the selection based on stability and trait performance.

Sum of *YSi* scores for each yield and contributing trait was computed to identify the overall ranking of genotypes and G6 scored highest followed by Swarna, G3, G14, G8, and G2. Overall ranking varied if we select among the contributing traits. The varieties which scored highest for DFF, DTM belong to late duration as the highest values were considered for calculation. Similarly for plant height, the tallest varieties scored highest *YSi* ranking. So selection of rank and its direction can be decided based on the requirements of target ecosystems. The ranking by *YSi* statistic based on predicted means for stability parameters for grain yield is shown in Table 5.

Table 5**Stability analysis of variance grain yield of genotypes across 3 environments**.

**Table:** 

	***d.f.***	**Sum of squares**	**Mean squares**	***F***	***p*.value**	**SS%**
TOTAL	44	3902.642				
GENOTYPES	14	1610.939	115.067	3.08	0.005	41.27816
ENVIRONMENTS	2	1245.561	622.7802	63.82	< 0.001	31.91583
INTERACTION	28	1046.142	37.3622	3.83	< 0.001	26.80601
HETEROGENEITY	14	229.0894	16.3635	0.28	0.988	5.870111
RESIDUAL	14	817.0531	58.3609	5.98	< 0.001	20.9359
POOLED ERROR	84		9.758

**Table:** 

**SL. NO**	**SPY**	**Mean yield**	**Yield rank**	**Adj.rank**	**Adjusted (Y)**	**Stability variance**	**Stability rating (S)**	**YSi (Y+S)**	**Superior lines**
G1	14_3	6.18	1	−3	−2	19.83	0	−2	
G2	148S	15.21	10	1	11	55.68**	−8	3	
G3	14S	20.87	15	3	18	29.39	0	18	√
G4	166_1	11.00	2	−2	0	88.049**	−8	−8	
G5	166_2	15.11	9	1	10	2.00	0	10	√
G6	166S	18.90	14	2	16	23.43	0	16	√
G7	248S	14.61	8	1	9	28.14	0	9	√
G8	24K	16.58	11	1	12	93.86**	−8	4	
G9	250K	13.02	6	−1	5	56.16**	−8	−3	
G10	3_1K	12.26	3	−1	2	−2.30	0	2	
G11	65S	12.96	5	−1	4	13.63	0	4	
G12	70S	12.63	4	−1	3	44.78*	−4	−1	
G13	75S	13.31	7	−1	6	50.25**	−8	−2	
G14	7K	16.67	12	1	13	44.01*	−4	9	√
G15	Swarna	18.13	13	2	15	13.50	0	15	√
Yield Mean		14.50							
Ysi Mean		4.93							
LSD		5.07							

* *P < 0.05*,

** P < 0.01.

### General genotypic adaptation

**Figure 2:**
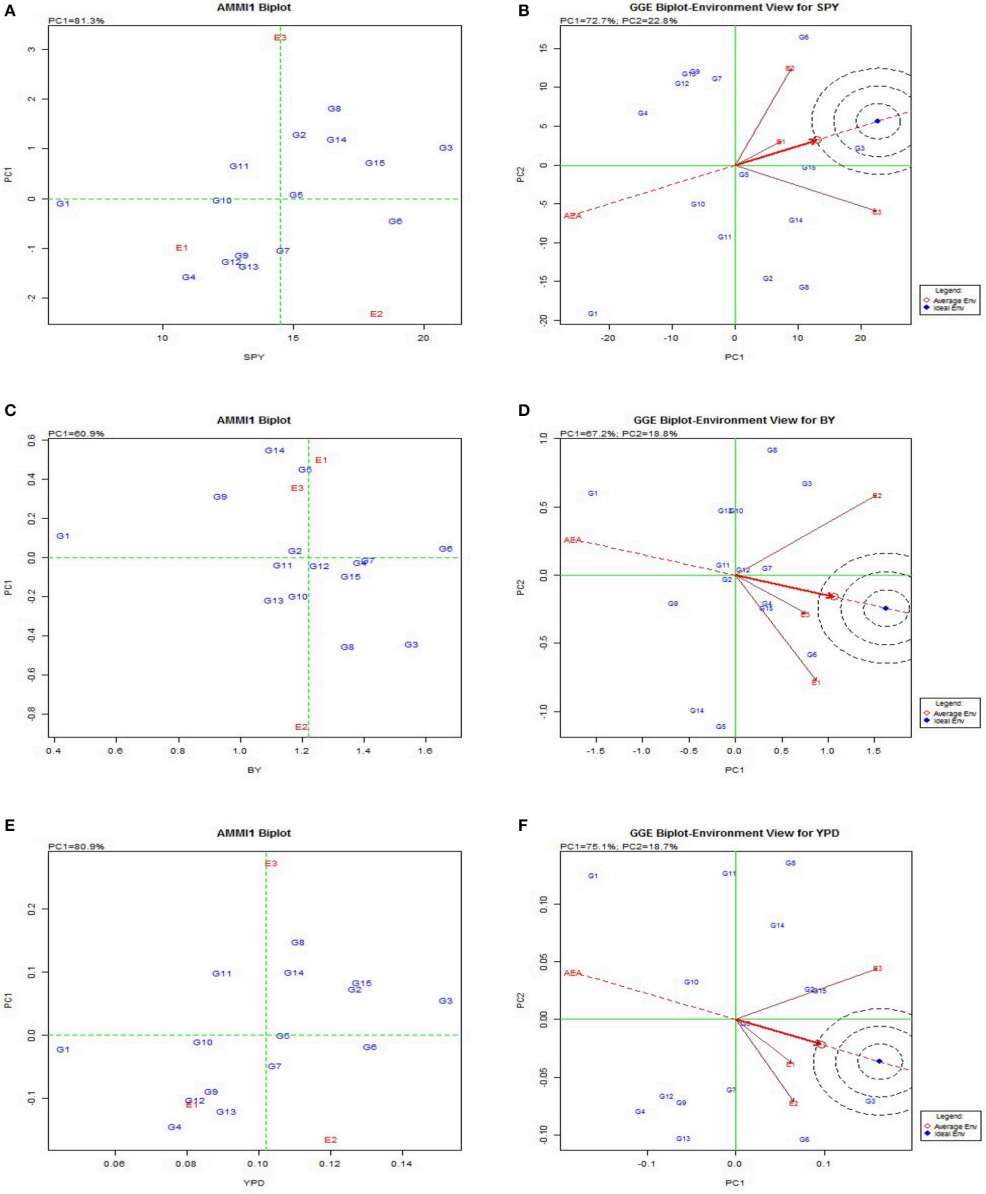
**AMMI and GGE biplot for the primary component of interaction (PC1) and mean yield(t/ha) or main effect of rice genotypes in differet seasons**. **(A)**, AMMI 1 biplot for single plant yield. **(B)**, GGE biplot for single plant yield. **(C)**, AMMI 1 biplot for bulk yield. **(D)**, GGE biplot for bulk yield. **(E)**, AMMI 1 biplot for per day productivity. **(F)**, GGE biplot for per day productivity.

AMMI and GGE biplot explained the general genotypic adaptation or stability across genotypes (Figure 2). To visualize the performance of different genotypes in a given environment, biplots were used. The relative ranking of different genotypes on the biplots is based on its projection onto the O-axis in AMMI Biplot and GGE biplot was used to diagnose the G × E interaction effects on each yield contributing trait. The results of the AMMI model analysis are interpreted on the basis of AMMI1 biplot where the graph is plotted with the main effect and first multiplicative axis term (PC1) for both genotypes and environments. Greater the Principal Component Axis (PC1) scores, either negative or positive, indicated the specific adaptation of a genotype to certain environments. The more the PC1 scores approximate to zero, the more stable the genotype among the environments under study. The AMMI biplot showed 81.3% fitness in the model for grain yield, and 60.9% for bulk yield. Among the BILs G8, G2, G3, G14, G11, and check Swarna (G15) exhibited high yield with high main (additive) effects showing positive PC1 score. BIL G10 showed less environmental interaction while three environments showed high interaction for GY. Consequently, for BY, *Kharif* 2014 (E2) showed high interaction but genotypes G2, G6, and G12 were identified with low environmental interactions and were considered best across the seasons for the trait. Based on AMMI analysis G10(3-1K) was the most stable genotype for BM; G6 and G3 for BY; G14 for DFF, DTM; G5 for FG, GN, TN; G3 for HI, GY, YPD; G2 for PH, TN, SF, 1000GW; G3 for PTN; G14 for TDM, TDMPD and G12 for TN. GGE biplot also showed similar results for stability of genotypes in trait expression across environments (Supplementary Figure 1). Genotypic variation was observed for each trait in case of adaptability to specific environments. *Kharif* environment was most favorable for high yielding BILs such as G3, G5, G2, and G14 while *Rabi* was favorable for G8 and G6 as they appeared most responsive for yield contributing traits in these respective environments. The environments E1 and E2 were more responsive for the traits BM, FG, GN, PL, PW, and SF and environment E3 was responsive for traits GY, TDM, YPD, TDMPD, DFF, DTM, and HI.

From the biplot graph of AMMI, it was inferred that interactions of environments are highly varied and all the three environments were highly interactive for most of the yield traits. E3 (*Rabi* season) appeared to be a favorable environment for BM and SF; E1 for BY, GN, PL, and E2 for PTN and GY. Genotypes G3(14S), G6(166S), and G9(250K) showed low interaction effects and hence they can be considered stable. In case of GY, G3, and G6 had high mean values and hence they can be recommended for all the environments. The genotypes with high interaction are suitable for specific environments, genotypes with high mean and positive interaction are suited for favorable environments and those with high mean and negative interaction are suited for unfavorable environments for the respective traits. A line that passes through the origin and is perpendicular to the O-axis in the biplots separates genotypes that yielded above the mean (G3, G4, G15, G7, G6, G8, G12, and G4) that would possibly yield above average in all the seasons and genotypes that yielded below average (G1, G14, G5, G10, G14, G9, and G1). The released varieties Tulasi, MTU1010, Swarna and Sahbhagi Dhan used as checks performed well across the seasons.

### Specific genotypic adaptation

**Figure 3:**
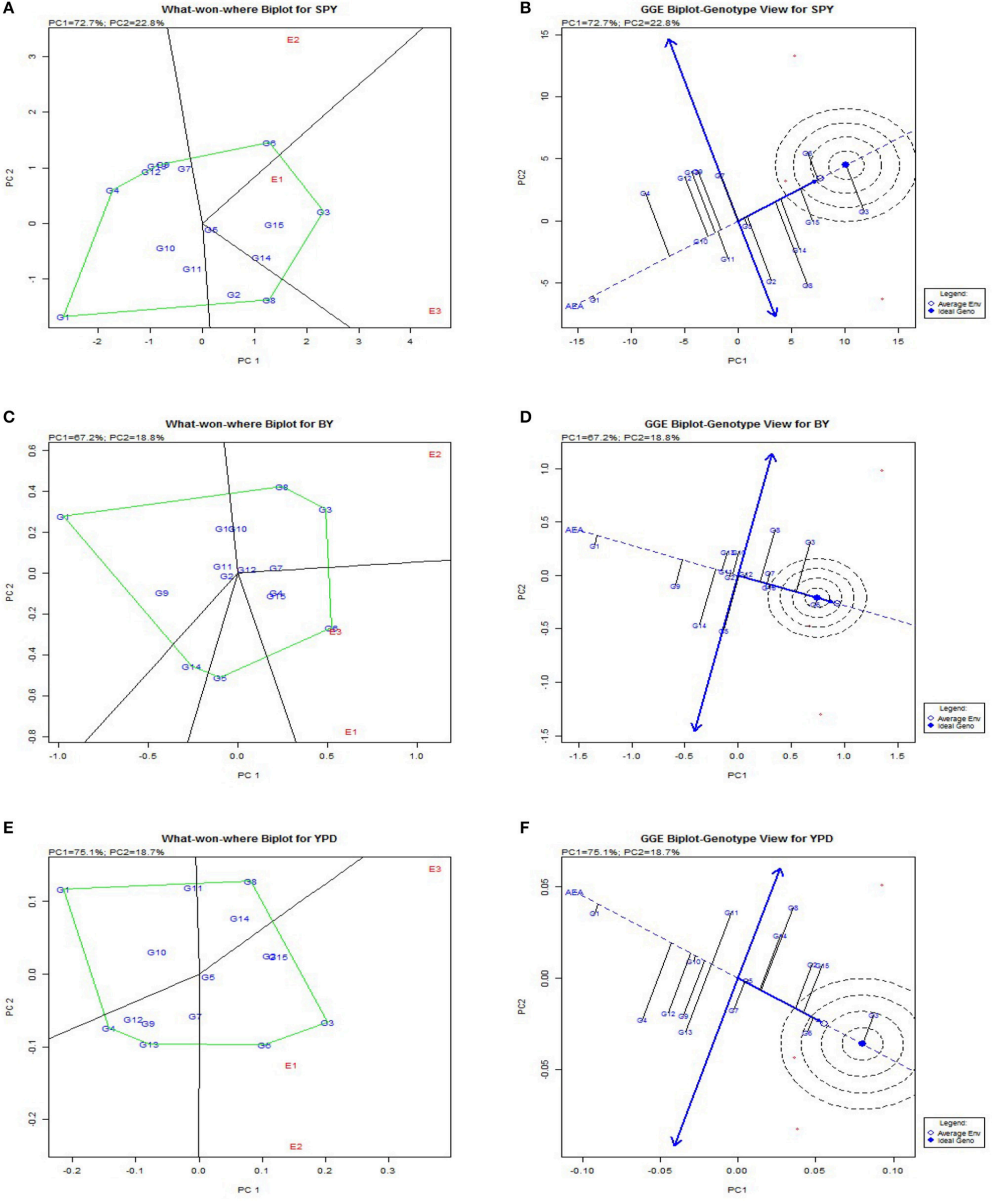
**Polygon views of the GGE biplot based on symmetrical scaling for “which-won-where” pattern of rice genotypes in three environments**. **(A)**, Polygon view of single plant yield. **(B)**, which-won-where plot single plant yield. **(C)**, Polygon view bulk yield. d which-won-where plot bulk yield. **(E)**, Polygon view per day productivity. **(F)**, which-won-where plot per day productivity.

Genotypic evaluation was conducted and based on GGE biplot which-won-where pattern and adaptation showed specific genotypic adaptation to limited environment conditions or the adaptability of genotypes for each environment (Figure 3). The same genotype performed best across three seasons for plant height (G2), 1000 grain weight (G2) (Supplementary Figure 2), harvest index (G3) and per day productivity (G3). This shows that these traits have stable expression across the seasons with limited environmental influence and the selected genotypes are most stable for the particular trait. The traits like biomass, days to flowering, days to maturity, filled grains, grain number, panicle weight, spikelet fertility, total dry matter, tiller number showed same genotype performed better in two *Kharif* (wet season) seasons under study but another genotype appeared to be best for *Rabi* (dry season). So these traits are showing seasonal variation and genotypic performance depends on environmental conditions. Traits like bulk yield, productive tiller number and grain yield showed no seasonal dependence on genotypic performance.

The polygon was drawn joining cultivars that are located farthest from the origin so that all other cultivars are contained in the polygon. Perpendicular lines to the sides of the polygon divide the biplot into sectors. Each sector has a vertex cultivar which is present in the corner of the polygon. The vertex cultivar is the best performing cultivar in the environments that share the sector with it. Vertex genotypes are G3, G4 and G7 at E1, E2 and E3 respectively. In case of checks, it is inferred that cultivar Sahbhagi Dhan is suited to *Rabi* season and Tulasi to *Kharif* season. The analysis indicated that G3, G12, and G4 were suitable BILs for cultivation in irrigated environment as they had the highest ranking in biplot and in predicted means.

### Association analysis

**Figure 4:**
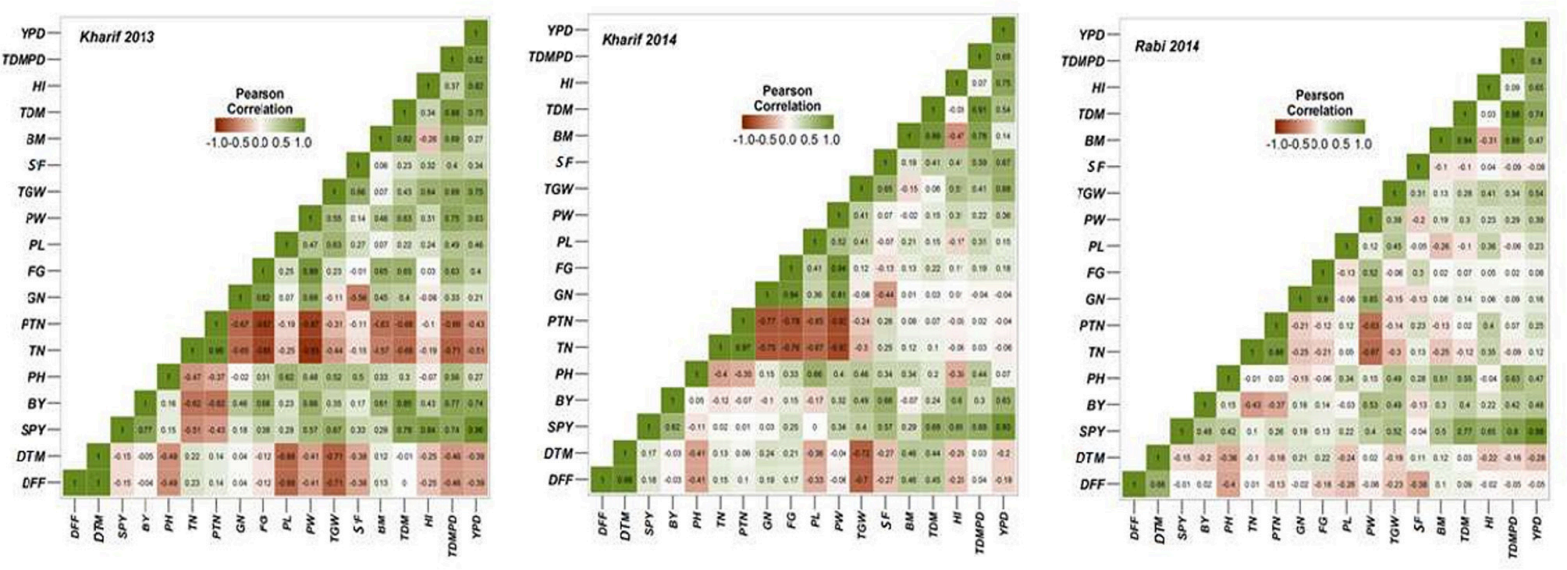
**Graphic representation of the correlation matrices of yield traits in three seasons**.

Multiple correlations between different yield and yield related traits was conducted for all the three seasons (Figure 4) and it was observed that grain yield has high significant association with panicle weight, 1000 grain weight, total dry matter, per day productivity and harvest index. Days to fifty percent flowering showed negative correlation with bulk yield, grain yield, spikelet fertility, 1000 grain weight, per day productivity and harvest index. Number of primary branches and secondary branches showed positive association both with filled and unfilled grains. Harvest index directly depended on grain yield, per day productivity, filled grains and panicle weight. In season wise correlation conducted among the yield traits, DFF showed highly significant association with DTM; TN with productive tiller number, panicle length and filled grain number; total number of grains with panicle weight in all the three seasons. Single plant yield showed stable and significant association with 1000 grain weight, biomass, harvest index and per day productivity across the seasons.

### Genotyping the BILs

**Figure 5:**
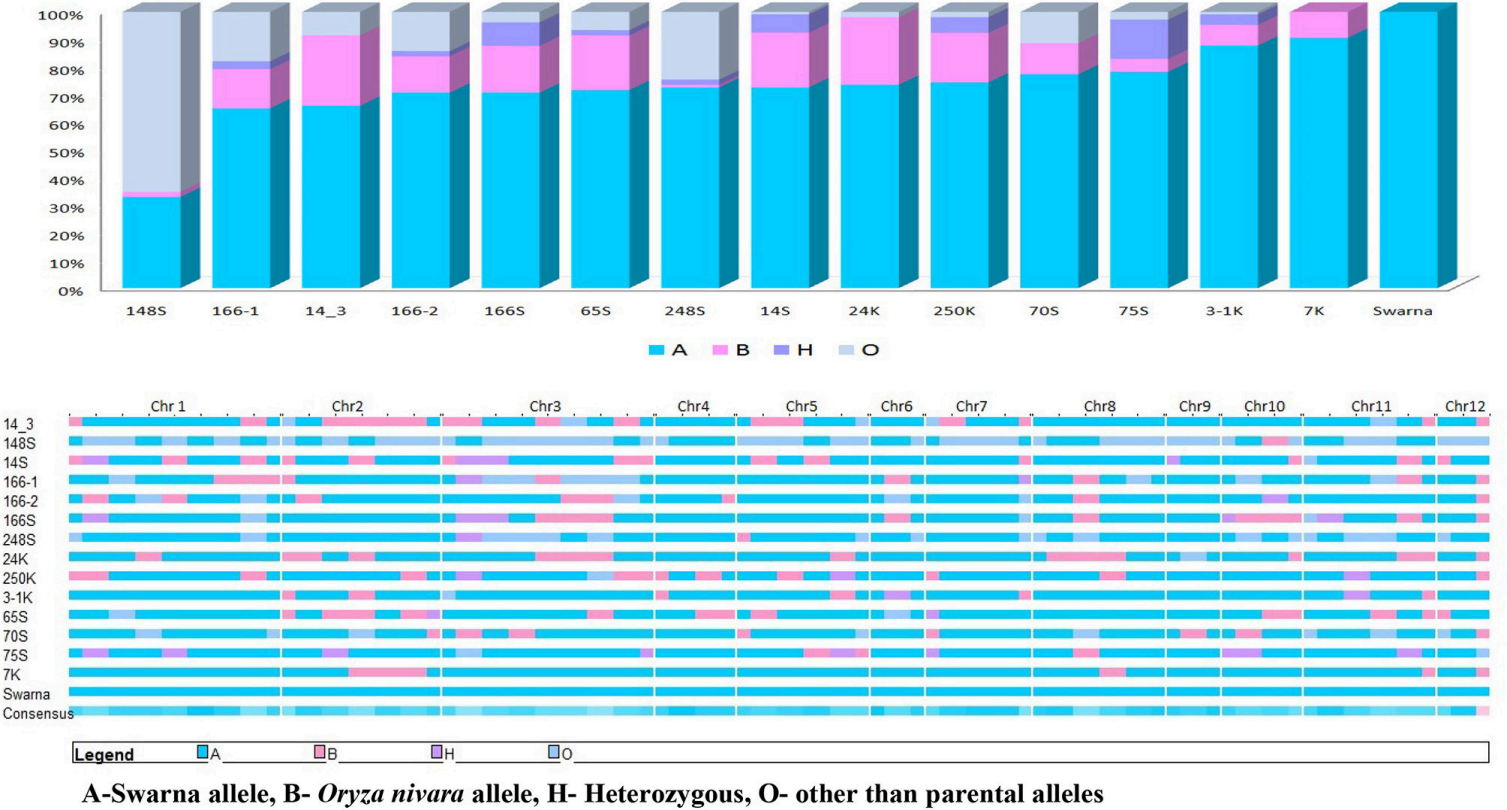
**Graphical representations of donor introgression in the backcross introgression lines using GGT software**.

All the BILs were screened using universal core genetic map for rice (Orjuela et al., 2010) and the genotypic data was analyzed using the Graphical Genotypes software (GGT 2.0) (van Berloo, 2008). 74 polymorphic SSR loci out of 165 genome wide core set microsatellite (SSR) markers were used for characterisation of BILs. On an average, percentage of recurrent genome of BILs varied from 36.8% (G2) to 90.6% (G14). The most stable and high yielding BILs, G6 (70.8%) and G3 (72.6%) had about 70% of recurrent parent genome and 20% donor parent genome with less than 10% of heterozygous segments. Further, G2 showed less percentage of recurrent parent genome and donor genome with maximum number of null alleles and recombination. Average percentage of recurrent parent genome in the genotypes was 74.7% and donor genome was 12.5%. Heterozygous segments average was 1.7% and null alleles were 9.7% and recombination was 18.5% (Figure 5).

Another panel of SSR markers which were linked to the reported QTLs from the same population was also used to screen these BILs. It was observed that many of the BILs have these reported QTLs in either homozygous or heterozygous condition. Yield QTL *yldp1.4* from *O. nivara* was present in most of the BILs.G6 had *O. nivara* allele of *yldp1.4* and its derived lines G4 and G5 had this QTL in completely heterozygous stage. G2 had four QTLs *yldp1.4 yldp2.3, nsp1.2* and *dtm 2.7* and G1 had three QTLs *yldp9.1, dtm9.3* and *nfg1.2*.

## Discussion

Pre-breeding and utilization of wild accessions are gaining importance in plant breeding programs for the identification of novel genes to improve yield levels of existing cultivars. Complex quantitative traits such as yield, with multiple contributing traits are highly influenced by environment interaction effects. Wide spread cultivation of rice in various agro ecological environments and the unpredicted effects of climate change makes the cultivation of stable and adaptable genotypes more desirable (Bose et al., 2012; Vanave et al., 2014). Stability and GEI studies are very important for the efficient breeding and adoption in multi-environment conditions (Kempton et al., 1997; Atlin et al., 2000; IRRI, 2006; Liang et al., 2015).

The yield of rice genotypes fluctuates considerably with change in environmental conditions (Bose et al., 2014). Segregation and appearance of wild traits in advanced generations are common phenomena in interspecific crosses. Keeping this in view, the present study was aimed at identification and characterization of a set of BILs developed from the same parental cross, for three seasons and stability was analyzed for key component traits for yield. Most of the multi location trials and multi-year testing focus only on the stability of grain yield but here we studied other yield contributing and component traits also.

Stability analysis models like *YSi* statistics, AMMI and GGE biplots are very useful in selecting lines with high homeostasis for broad target environments and were utilized in multilocation trials and in coordinated variety testing programmes. Prasad et al. (2001) studied stability and yield performance of mega varieties using the data from All India Coordinated Rice Improvement Programme (AICRIP) as well as international trials for a period of 25 years and identified four mega environments for testing the varieties for yield potential in India. Many studies have used GGE biplot analysis mainly for mega-environment evaluation, cultivar evaluation, and assessment of varietal stability (Kang, 1993; Yan and Hunt, 2001; Yan and Kang, 2003; Dehghani et al., 2006; Navabi et al., 2006; Blanche et al., 2007; Ding et al., 2007; Jalata, 2011; Mohammadi et al., 2012; Rakshit et al., 2012; Amiri et al., 2015). Balestre et al. (2010); Nassir (2013) studied stability and adaptability of upland rice genotypes by the GGE biplot method based on the predicted genotypic and phenotypic values. The simultaneous selection for high mean and stability results in the selection of superior genotypes with non significant stability variance and it enhances quality of selection. This method was successfully utilized in most of the crops including rice (Wade et al., 1999; Ouk et al., 2007; Tariku et al., 2013) especially for assessing grain yield.

In this study, three seasons data was subjected to correlation analysis and the traits which are associated significantly were discussed for stability analysis. Stability analysis models helped identification of superior genotypes with both high mean yield and stability. Different stability analysis models showed that G3 is the most stable genotype for grain yield followed by G6 and Swarna. G5 and G6 were identified to be stable and ideal genotypes for bulk yield, grain number and number of filled grains, followed by G3, G7, G15, G8, and G12. G14 was identified as most stable genotype for biomass, flowering duration, panicle length and total dry matter production. G2 showed stability and high mean value for 1000 grain weight, spikelet fertility, plant height and panicle length; it was identified as stable genotype for days to fifty percent flowering and days to maturity with lowest mean value indicating the most stable short duration BIL followed by G3. Some of the superior genotypes for yield specific traits with less stability across the seasons can be stabilized with limited back cross approach (Singh and Huerta-Espino, 2004) with an adapted cultivar.

An ideal genotype would be one that has both high mean yield and high stability. The position of an “ideal” genotype is closer to the direction of the mean environment and has a zero projection onto the perpendicular AEC ordinate. G2 and G4 showed high mean ranking and were identified as the best performing lines in terms of both mean yield and stability across environments in the irrigated ecosystem. Based on adaptation map G2 is best adapted for environment E1, G4 for E2 and G7 for E3. Response plot for mean yield also indicated the same results across the seasons.

**Figure 6:**
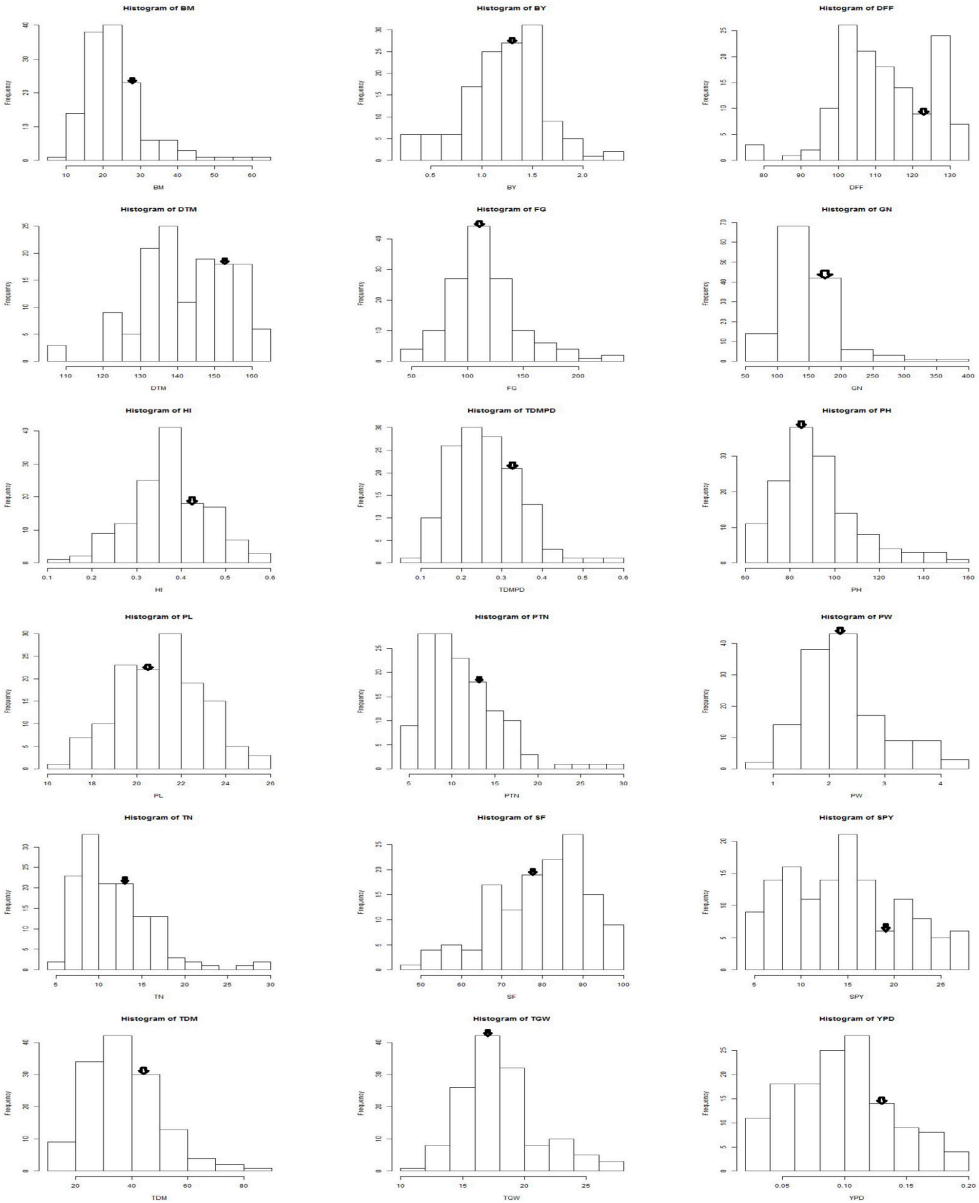
**Frequency distribution of yield and yield related traits in Swarna / ***O. nivara*** derived BILs arrow indicates Swarna mean value**.

Among the genotypes recurrent parent Swarna was stable across the seasons for traits like bulk yield, biomass, days to maturity, number of filled grains, panicle length, productive tiller number, panicle weight. The mega variety Swarna, which is popular in major rice growing countries like India, Bangladesh, Philippines and Thailand; is known for its adaptability in wide range of environments (Prasad et al., 2001). The performance of the BILs was on par or above the mega varieties like Swarna and most popular cultivars of different durations. As the BILs and checks belong to different maturity groups, per day productivity (YPD) was considered to compare their yield. The frequency distribution of the pooled data of three seasons for each trait followed a bell shaped curve with Swarna placed on the peak of the curve (Figure 6). However, G6 and G3 proved significantly superior in yield over the recurrent parent Swarna and on par with the best check MTU1010. Graphical representation of the molecular marker data has relevance in studying the genome constitution of the recombinant population (Young and Tanksley, 1989). It was observed that the BILs had more than 70% of recurrent parent genome. Tian et al. (2006) reported that the high-yielding ILs contained relatively less introgressed segments than the low-yielding ILs in a set of 159 ILs derived from *Oryza rufipogon* in *indica* cultivar Guichao2 back ground using 126 polymorphic SSRs.

**Figure 7:**
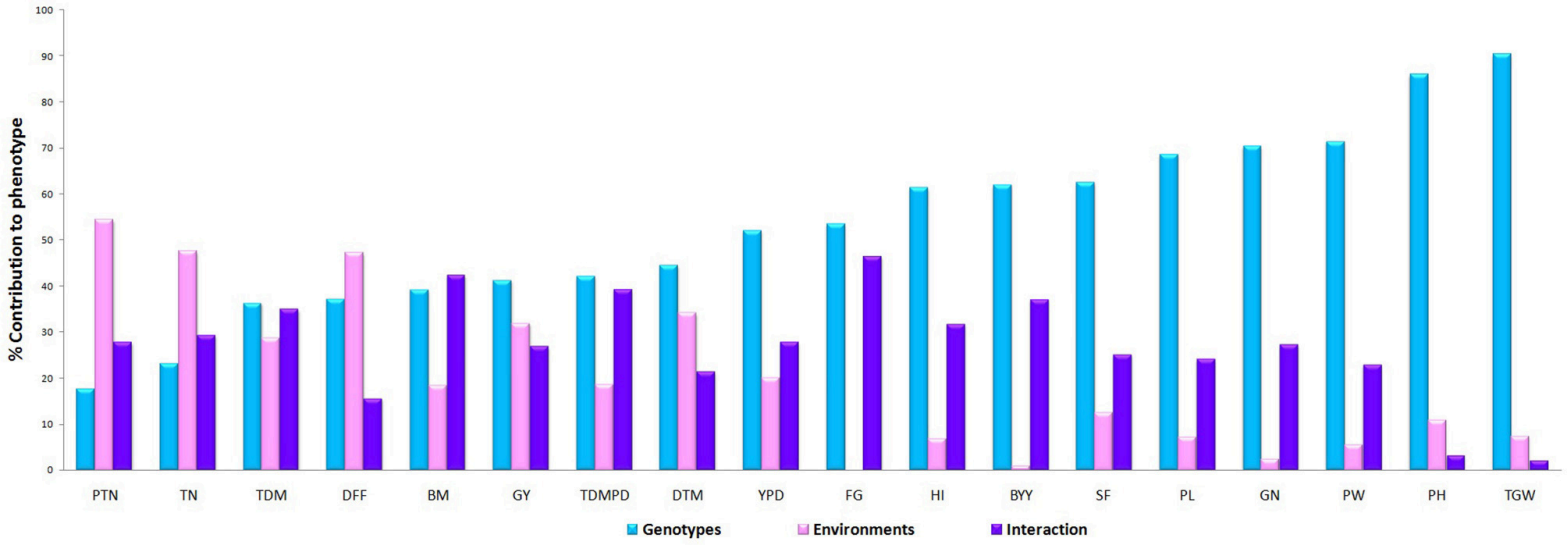
**The factor explained SS(%) was calculated comparing sum of square (SS) from AMMI ANOVA showing the percentage contribution of genotype environment and interaction effects in phenotypic expression of each trait across environments**.

**Figure 8:**
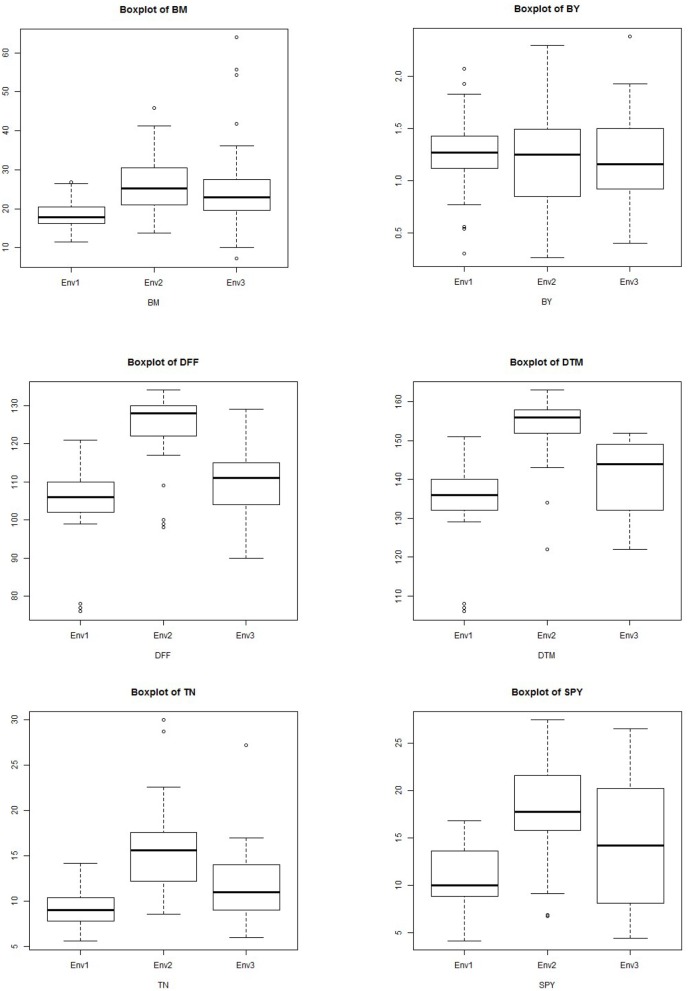
**Boxplot showing the differences in yield and related traits among subpopulations**. Box edges represent the upper and lower quantile with median value shown as bold line in the middle of the box. Whiskers represent 1.5 times the quantile of the data. Individuals falling outside the range of the whiskers shown as open dots.

Plant height and 1000 grain weight were the most stable traits across the season with minimal genotypic variation and with PC1values of 96.5% and 97.8% respectively in GGE biplot (Figure 7). The explained SS (%) factor was calculated comparing sum of square (SS) from AMMI ANOVA showing the percentage contribution of genotype, environment and interaction effects in phenotypic expression of each trait. It was observed that grain yield was contributed mainly by genotype (41.28%), followed by environment (31.92%), and their interaction (26.81%). The percentage of explanation of phenotype by genotypic contribution was high for 1000 grain weight (90.40%) and plant height (85.99) and environment effect was high for tiller number (47.56), days to flowering (47.24) and grain yield (31.92) while interaction effect was high for bulk yield and filled grains per panicle (Figure 8).

The G × E interactions for most of the yield traits under study were significant but some traits showed stable genotypic performance across the environments. The seasonal variation between the highest value and lowest value was observed for traits BM, BY, DFF, DTM, TN, and GY but the difference was minimal for traits GN, PL, PW, TGW, SF, HI, and YPD (Supplementary Figure 3). For the widely varying traits with high GEI, additional agronomic management is also required along with crop improvement for trait stability. Crossover GE and dissimilarity between environments for discriminating genotypes were very low in case of GN, PH, PL, PW, SF, and TGW but were moderate in case of all other traits. Identification of stably expressing contributing traits is very essential for crop improvement for any major trait than combining multiple traits which fluctuate across the environments. It was observed that 1000 grain weight is the trait contributing most to yield and is stably inherited as well as stably associated with grain yield. So while considering the grouping of the genotypes for multi location yield trials, this trait should also to be considered along with duration for a reliable comparison and analysis. The future breeding programs need to focus on improvement of stably performing traits with high heritability to develop stable high yielding genotypes. Dalvi et al. (2007), Panwar et al. (2008), Waghmode and Mehta (2011), and Padmavathi et al. (2013) studied the GE interaction for grain quality in rice and the stability of grain quality is also important in multilocation trials if quality is the selection criteria.

In this study, we used two *Kharif* and one *Rabi* season data and there was significant seasonal variation observed for most of the traits. In case of yield GEI were higher in *Kharif* season than in *Rabi* season and similar results were reported by Atlin et al. (2000). GGE biplots indicated that *Kharif* 2013 was the ideal season to select genotypes for BM, HI, PL, PTN, SF, GY, TDM, TGW, *Kharif* 2014 for DFF, FG, GN, PH, PW, and *Rabi* 2014 for BY and TGW. *Kharif* seasons were most ideal seasons to select BILs for yield contributing traits; especially *Kharif* 2014 was discriminative as well as representative among the seasons and was suitable for selecting genotypes with general adaptation. *Rabi* season was the most discriminating and least representative environment for testing genotypes and is useful in selecting only specifically adapted genotypes. *Kharif* 2013 was found least discriminating but most representative among the seasons for most of the traits. The highest environmental averages for all the yield traits were observed in either of the two *Kharif* seasons. The maximum value was also observed in the *Kharif* seasons except for traits like SF, BM, and YPD which showed maximum value in *Rabi*. The significant difference due to environment indicated the existence of genotypic differences in adaptability. Genotypes also differed considerably with respect to their stability for yield traits. Similar observations on GEI were made by Gauch and Zobel (1996); Wade et al. (1999); Ouk et al. (2007); Das et al. (2009); Sreedhar et al. (2011); Tariku et al. (2013); Akter et al. (2014) on multi environment studies using rice genotypes. All the three models showed similar results and utilization of *Ysi* statistic is advantageous and complements the AMMI and GGE method for selecting stable and high yielding genotypes (Nassir and Ariyo, 2011). Kumar et al. (2012) used different models for stability analysis and the correlations between the stability rankings of entries produced by the GGE model and the parameters of Shukla, AMMI, showed very high rank correlation coefficients.

Advanced BILs with stable yield traits can be grown in several environments to study QTL x environment interactions and these lines can be used in breeding programmes as well as to develop varieties in relatively less duration (Jeuken and Lindhout, 2004). Further studies will focus on (i) development of BIL x BIL mapping population from the stable identified genotypes (ii) identification of QTL for stable contributing traits for yield and (iii) development of varieties from selected stable BILs through multi location variety trials.

## Conclusions

The study showed the importance of genotype × environment interaction and stability analysis for evaluation of genotypic yield potential. Wild introgression lines derived from *O. nivara* in Swarna background were studied for stability for yield related traits in *Kharif* and *Rabi* season. The stability and adaptability studies using AMMI, GGE biplot and *Ysi* statistics indicated G3(14S) and G6(166S) as the most stable BILs with high yield performance. The percentage of explanation of genotype on phenotype was high for 1000 grain weight and plant height and environment effect was high for tiller number, days to flowering and grain yield and interaction effect was high for bulk yield and filled grains per panicle. DRR Dhan 40, an elite BIL and recently released variety showed yield stability with high mean performance and the mega varieties which were used as checks also showed yield stability across the seasons. It was observed that wild derived lines with about 70% of recurrent parent genome were more stable and showing enhanced yield levels. Thus, more emphasis should be devoted in future breeding programs to pre breeding and to develop genotypes with wider adaptation. Stability analysis and GEI may be further extended widely for stress resistance, quality as well as nutrient composition for precise identification of superior genotypes.

## Ethics statement

The authors declare that the experiments comply with the current laws of the country in which they were performed and in compliance with ethical standards.

## Author contributions

DB, SN conceived and planned the work. Phenotypic and genotypic screening was performed by JB, AR, RY, KB, SM, MS, DB, RP, and GP. DS, DB, analyzed the data. DB wrote the manuscript. DS and SN revised and proofread the manuscript. Facilities provided at Indian Institute of Rice Research by VB.

### Conflict of interest statement

The authors declare that the research was conducted in the absence of any commercial or financial relationships that could be construed as a potential conflict of interest.

## Abbreviations

AMMI: additive main effect and multiplicative interaction
G × E: genotype by environment
GGE: Genotype and Genotype × Environment Interaction
BILs: backcross introgression lines
GEI: Genotype Environment Interaction
AICRIP: All India Coordinated Rice Improvement Programme
PCA: principal component analysis
IIRR: Indian Institute of Rice Research
DFF: days to fifty percent flowering
DTM: days to maturity
PH: plant height
TN: tiller number
PTN: number of productive tillers
PL: panicle length
PW: panicle weight; FG number of filled grains
TG: total number of grains
TGW: 1000 grain weight
GY: grain yield
BM: biomass
SF: spikelet fertility
TDM: total dry matter
HI: harvest index
TDMPD: total dry matter per day
YPD: per day productivity
BY: bulk yield.
