# Supplementary material for: Genotype × Environment Interactions of Yield Traits in Backcross Introgression Lines Derived from Oryza sativa cv. Swarna/Oryza nivara
Source: Front Plant Sci. 2016 Oct 19;7:1530. doi: 10.3389/fpls.2016.01530 (PMC5070172; doi:10.3389/fpls.2016.01530)

Supplementary Fig.1 AMMI and GGE biplot for the primary component of interaction (PC1) and mean yield(t/ha) or main effect of rice genotypes in differet seasons

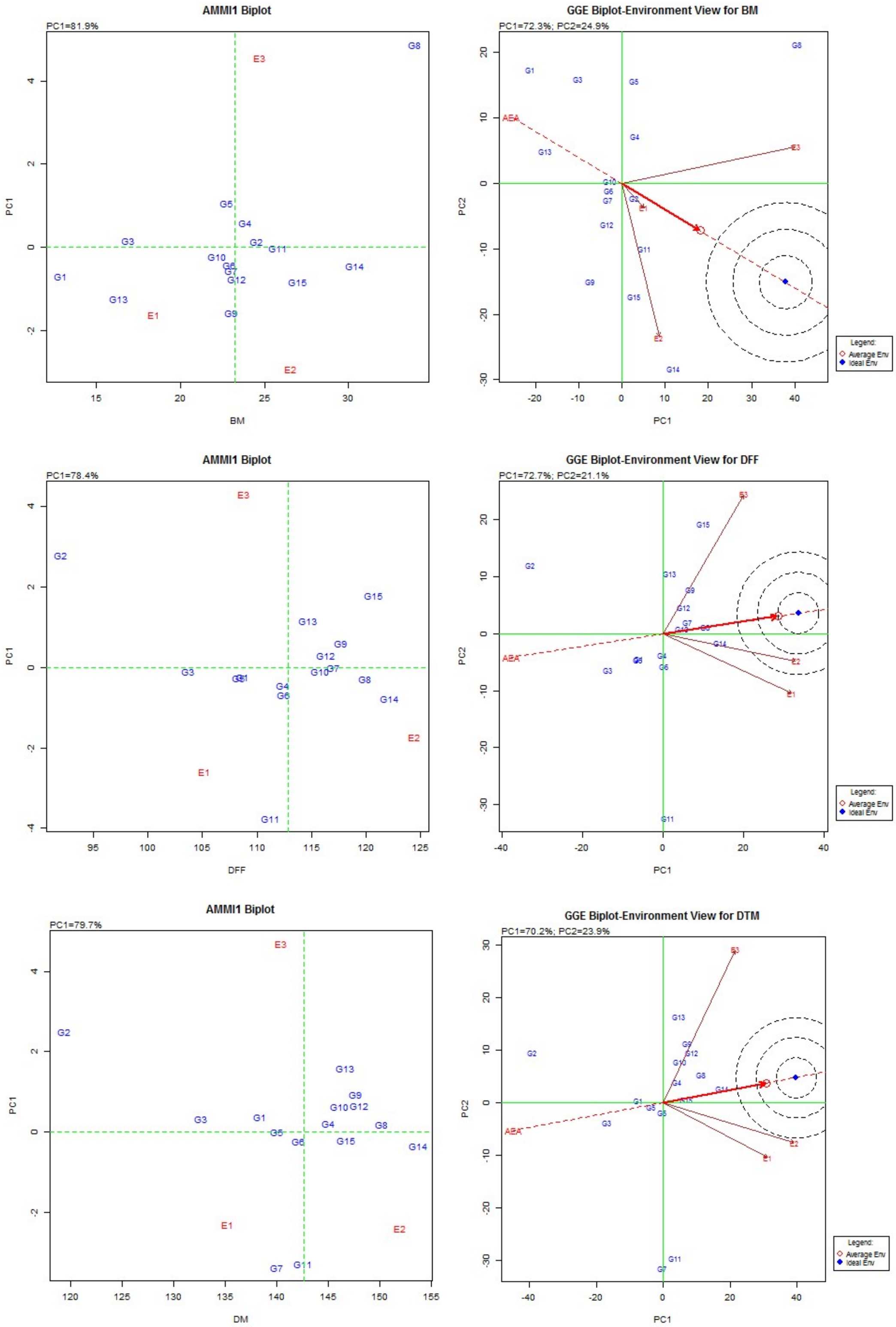

Supplementary Fig.1 AMMI and GGE biplot for the primary component of interaction (PC1) and mean yield(t/ha) or main effect of rice genotypes in differet seasons

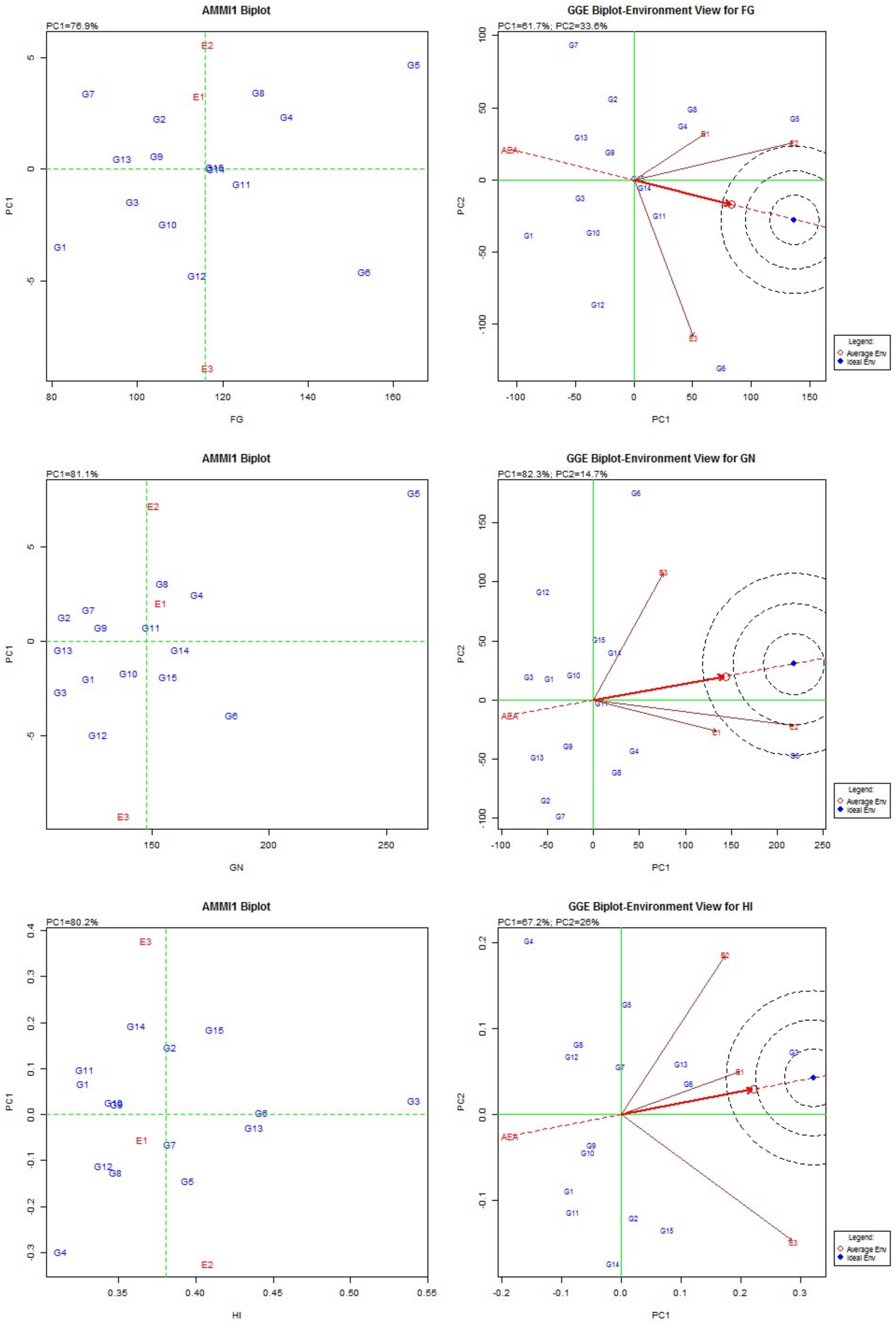

Supplementary Fig.1 AMMI and GGE biplot for the primary component of interaction (PC1) and mean yield(t/ha) or main effect of rice genotypes in differet seasons

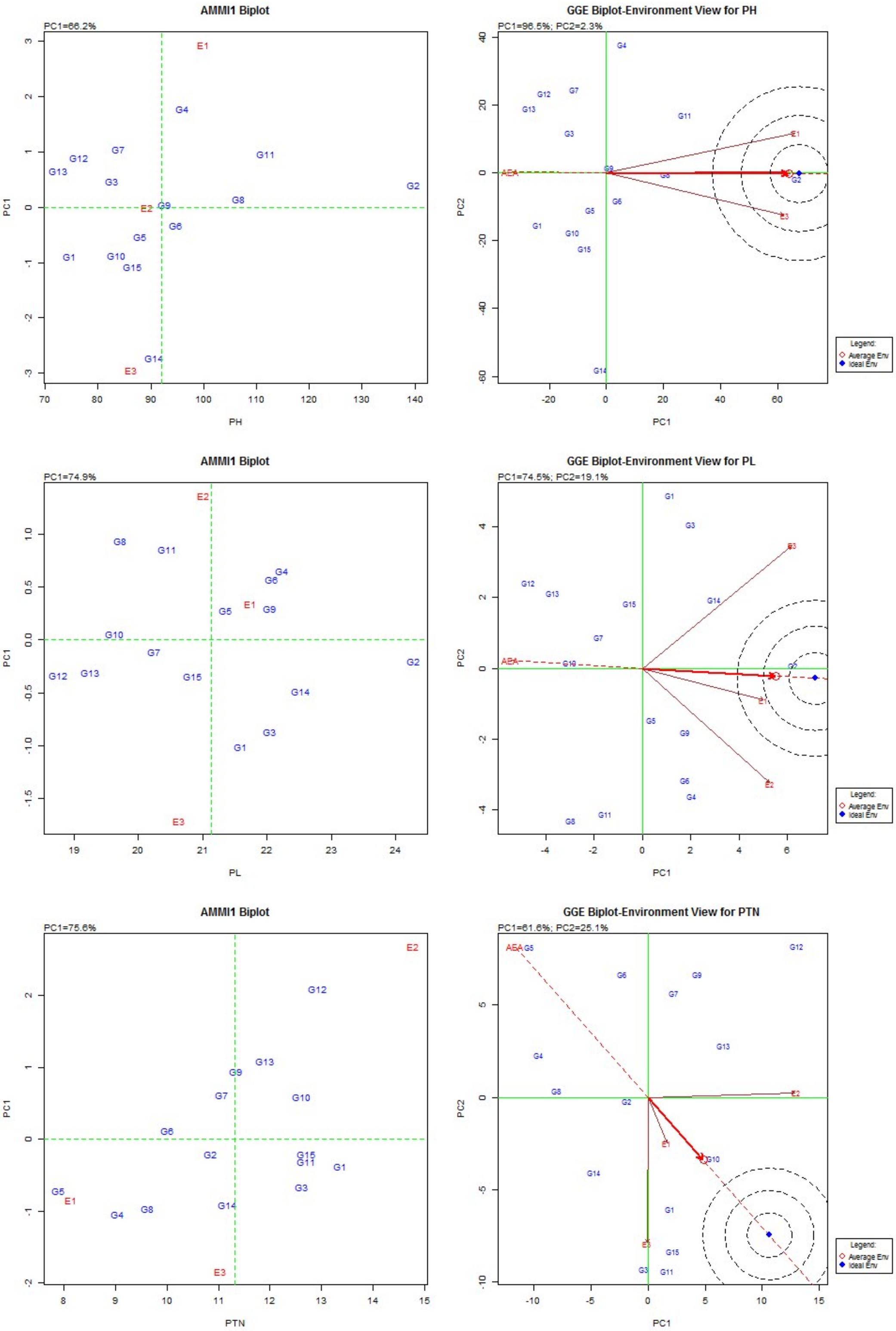

Supplementary Fig.1 AMMI and GGE biplot for the primary component of interaction (PC1) and mean yield(t/ha) or main effect of rice genotypes in differet seasons

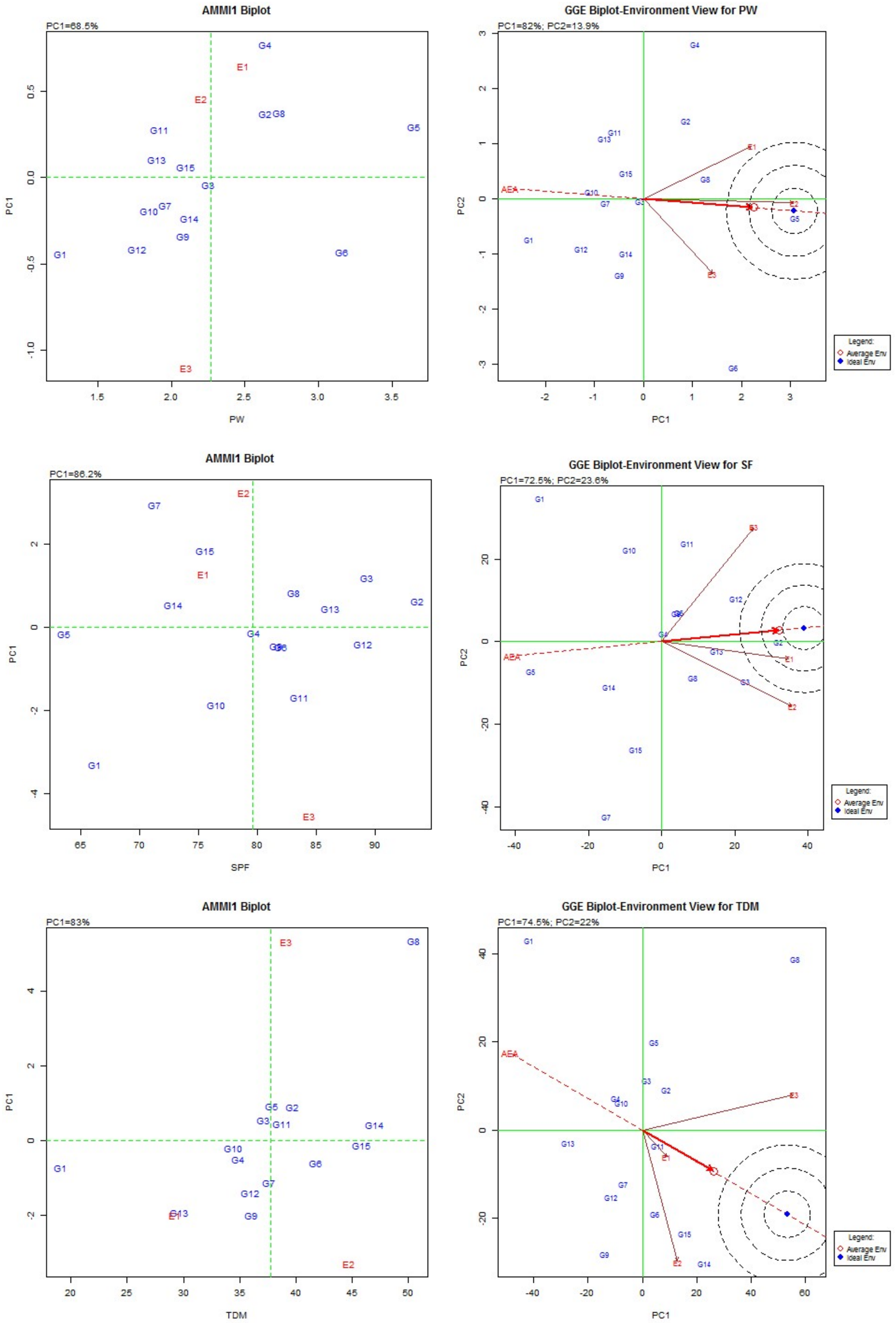

Supplementary Fig.1 AMMI and GGE biplot for the primary component of interaction (PC1) and mean yield(t/ha) or main effect of rice genotypes in differet seasons

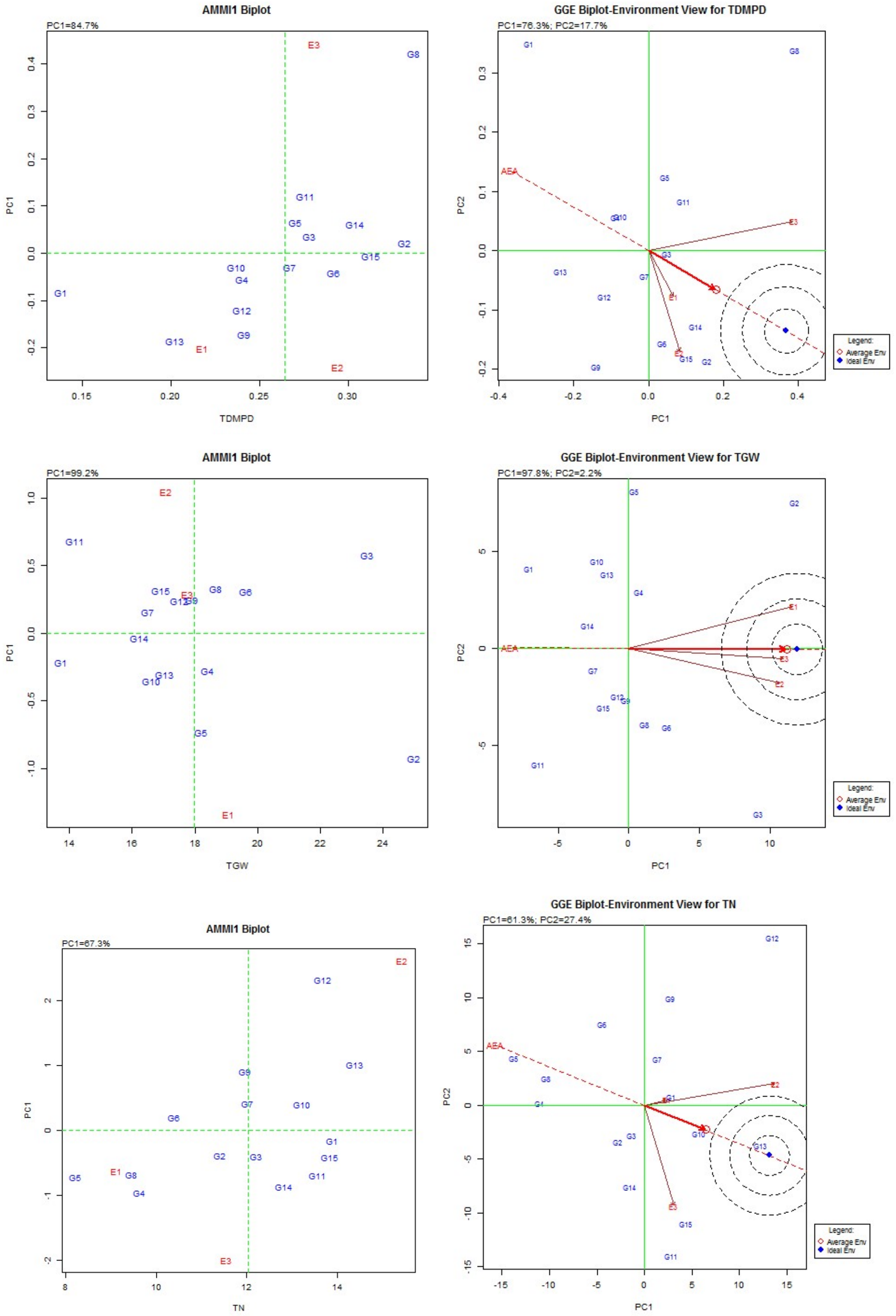

Supplement: Supplementary file 1 [file DataSheet1.pdf]
