# Supplementary material for: Genotype × Environment Interactions of Yield Traits in Backcross Introgression Lines Derived from Oryza sativa cv. Swarna/Oryza nivara
Source: Front Plant Sci. 2016 Oct 19;7:1530. doi: 10.3389/fpls.2016.01530 (PMC5070172; doi:10.3389/fpls.2016.01530)

Supplementary Fig.2 Polygon views of the GGE biplot based on symmetrical scaling for 'which-won-where' pattern of rice genotypes in three environments

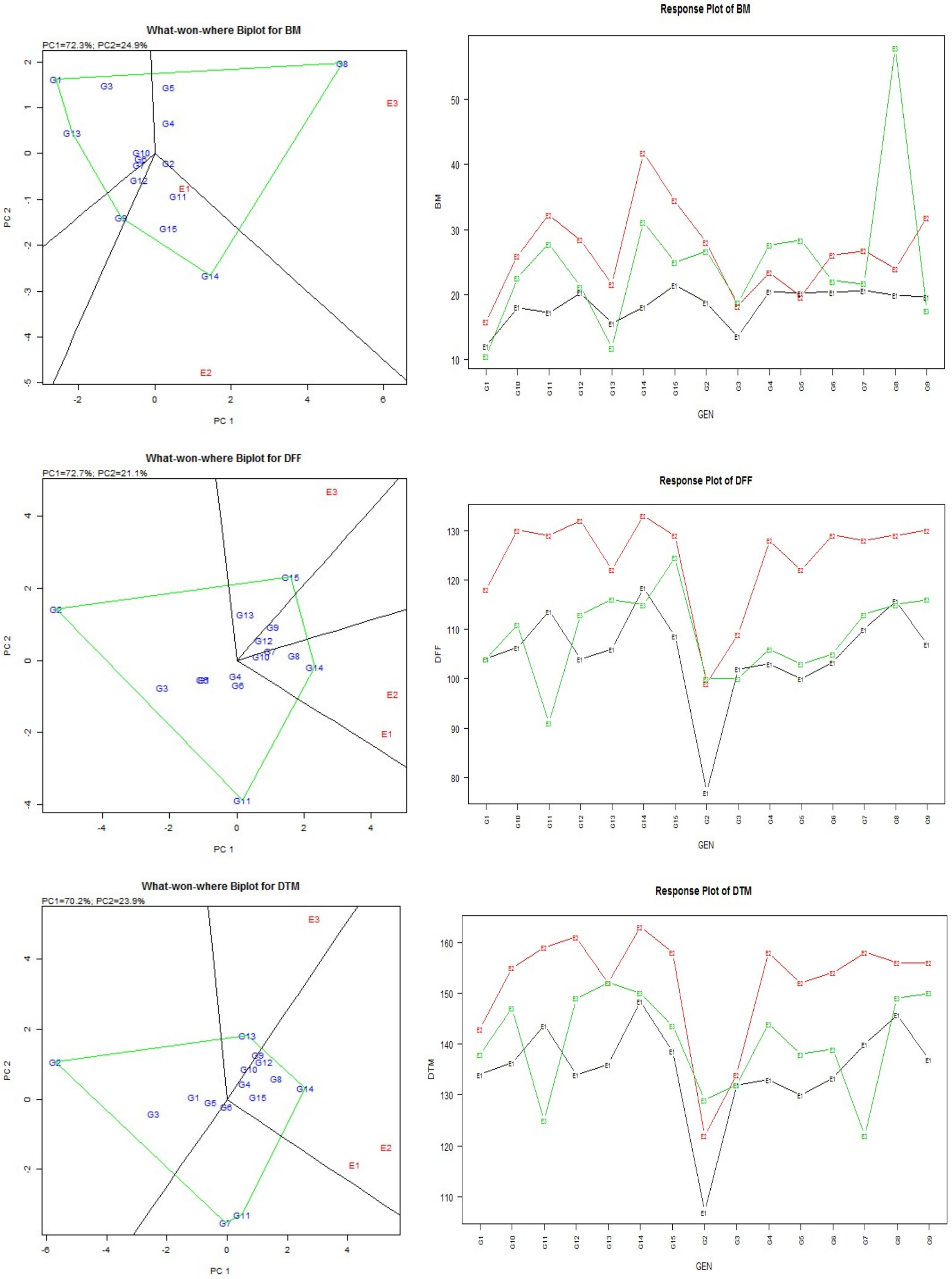

Supplementary Fig.2 Polygon views of the GGE biplot based on symmetrical scaling for ‘which-won-where’ pattern of rice genotypes in three environments

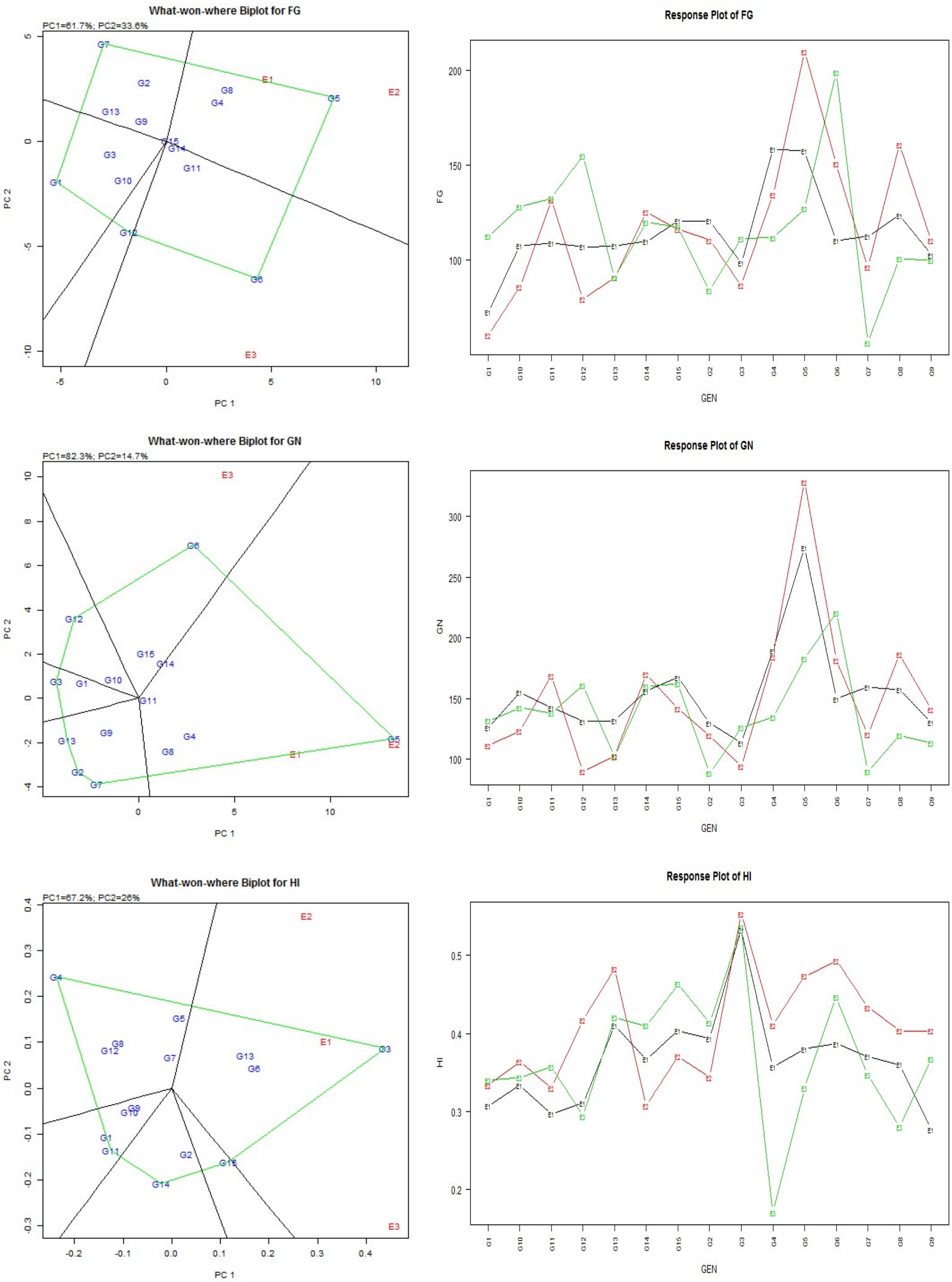

Supplementary Fig.2 Polygon views of the GGE biplot based on symmetrical scaling for 'which-won-where' pattern of rice genotypes in three environments

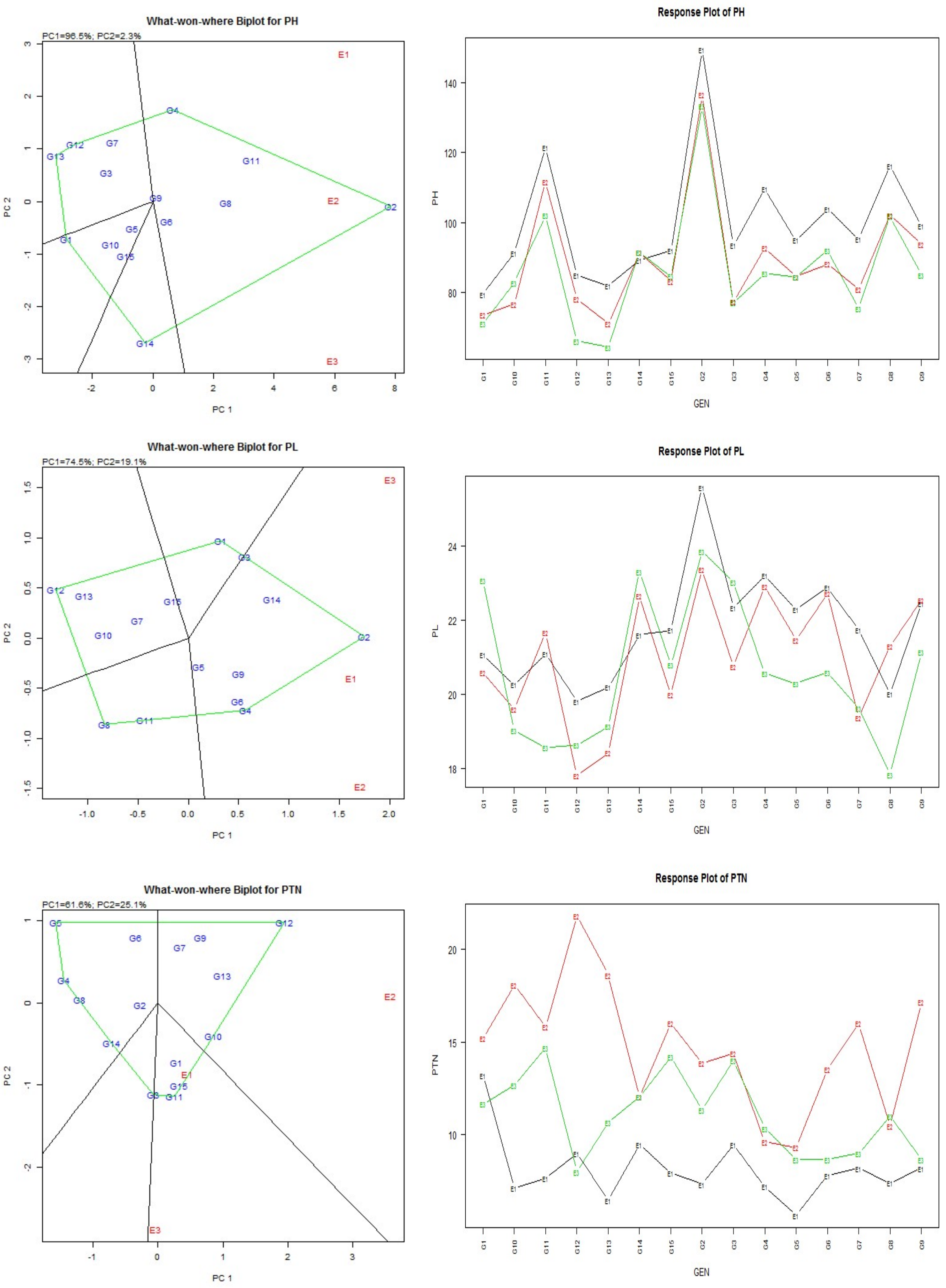

Supplementary Fig.2 Polygon views of the GGE biplot based on symmetrical scaling for ‘which-won-where’ pattern of rice genotypes in three environments

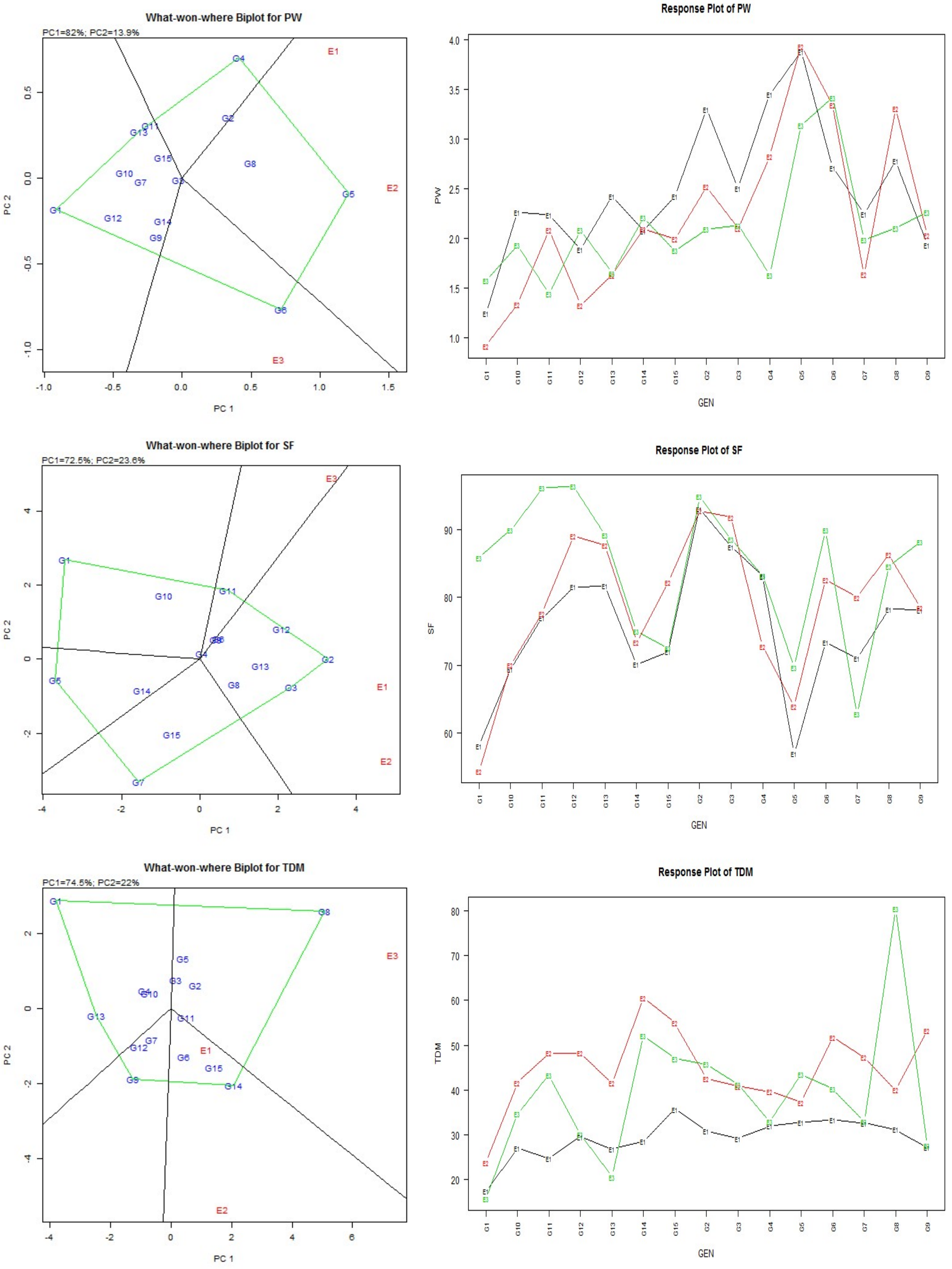

Supplementary Fig.2 Polygon views of the GGE biplot based on symmetrical scaling for 'which-won-where' pattern of rice genotypes in three environments

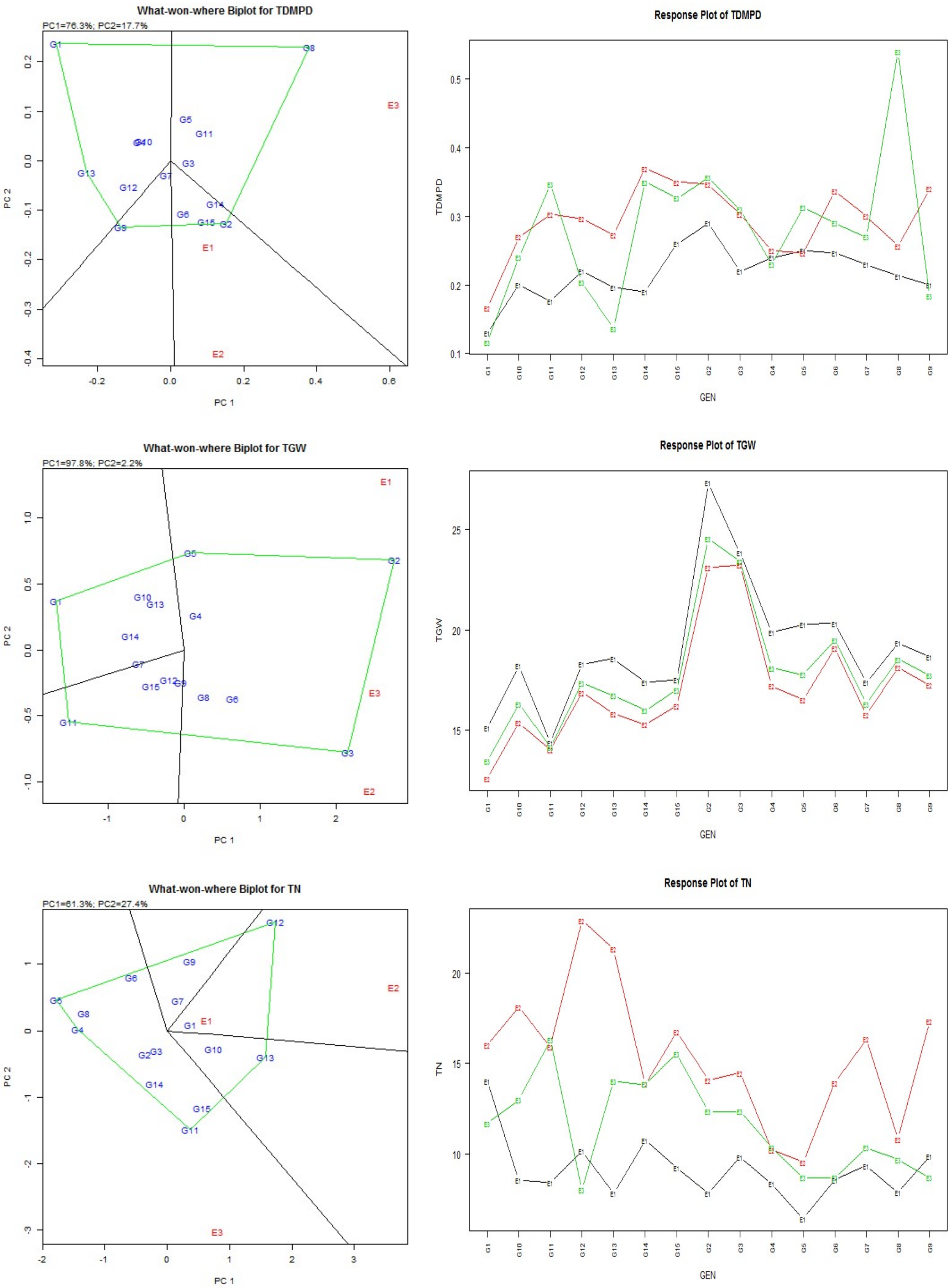

Supplement: Supplementary file 2 [file DataSheet2.pdf]
