# Supplementary material for: Genotype × Environment Interactions of Yield Traits in Backcross Introgression Lines Derived from Oryza sativa cv. Swarna/Oryza nivara
Source: Front Plant Sci. 2016 Oct 19;7:1530. doi: 10.3389/fpls.2016.01530 (PMC5070172; doi:10.3389/fpls.2016.01530)

Supplementary Fig.3 Box plot representation of genotypic performance across seasons

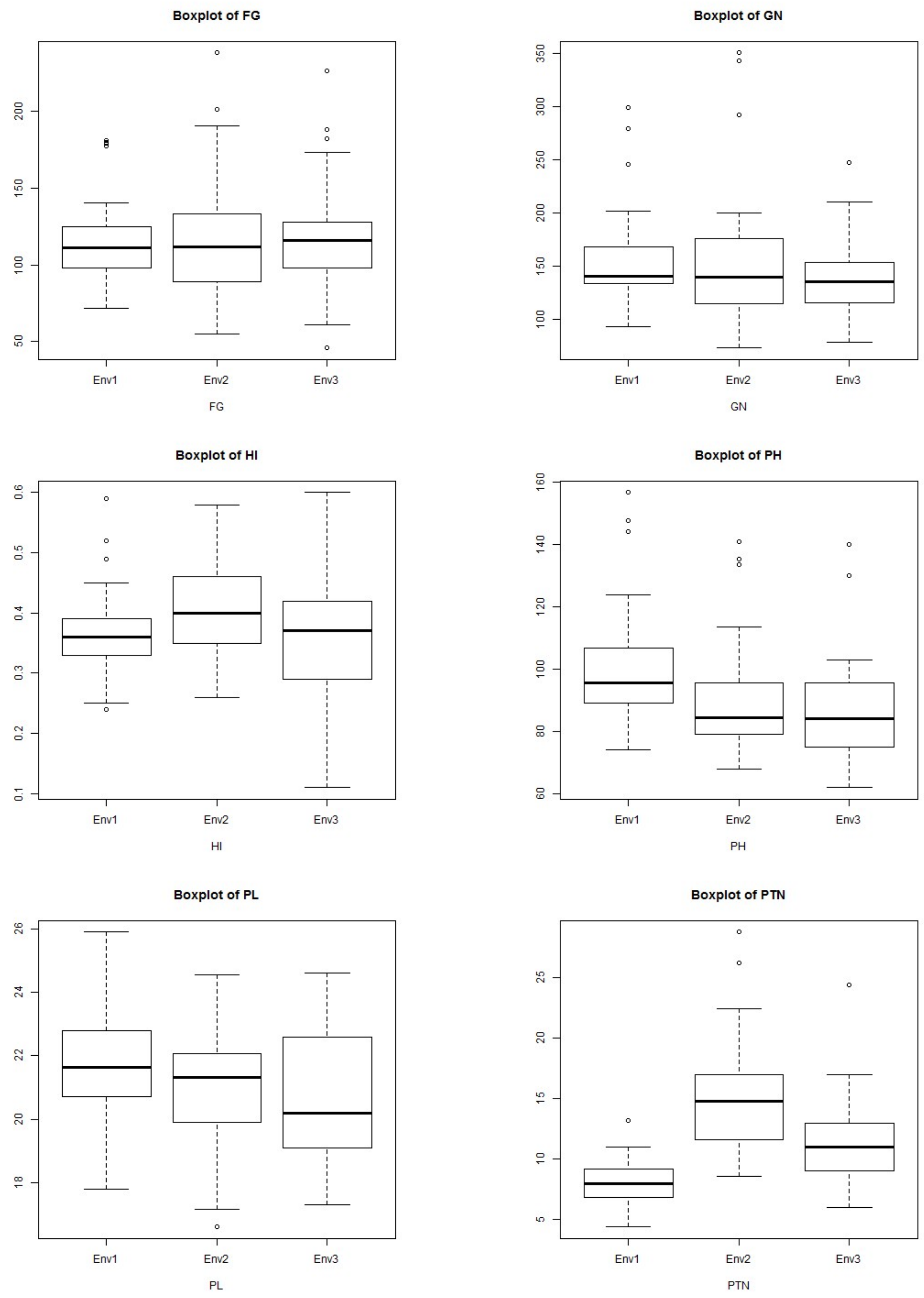

Supplementary Fig.3 Box plot representation of genotypic performance across seasons

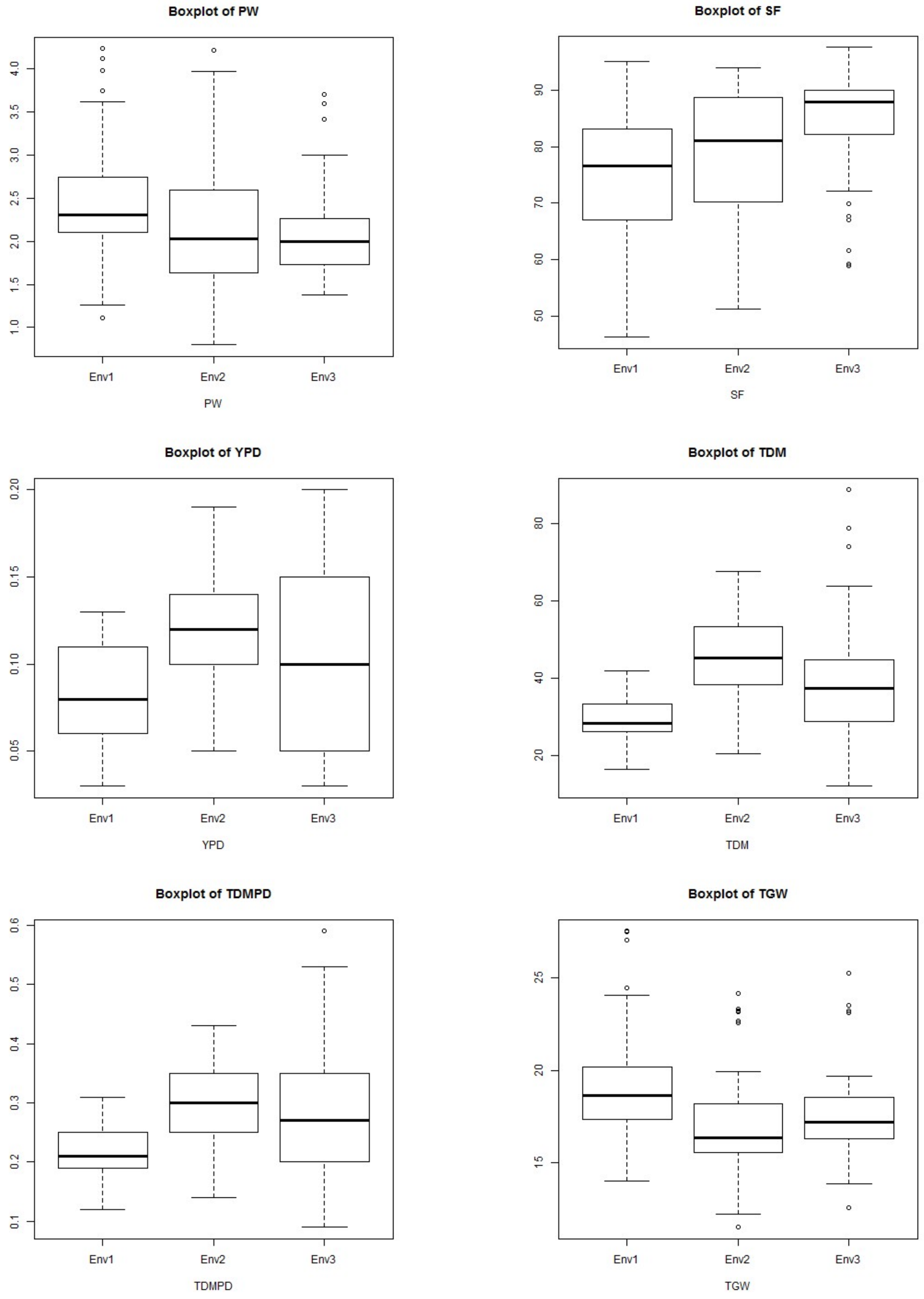

Supplement: Supplementary file 3 [file DataSheet3.pdf]
