# Supplementary material for: Genotype × Environment Interactions of Yield Traits in Backcross Introgression Lines Derived from Oryza sativa cv. Swarna/Oryza nivara
Source: Front Plant Sci. 2016 Oct 19;7:1530. doi: 10.3389/fpls.2016.01530 (PMC5070172; doi:10.3389/fpls.2016.01530)
Supplement: Supplementary file 4 [file DataSheet4.pdf]

Supplementary table 1 . List of polymorphic markers used in the study

SSR	Chromosome number	Forward primer	Reverse primer	Repeat motifs	Size	Position (CM)	Anneal Temperature
RM 5	1	TGCAACTTCTAGCTGCTCGA	GCATCCGATCTTGATGGG	(GA)14	113	117.8	55
RM9	1	GGTGCCATTGTCGTCCTC	ACGGCCCTCATCACCTTC	(GA)15GT(GA)2	136	92.4	55
RM495	1	ATGATGATGGACGACGACAACG	TGAATCCAAGGTGCAGAGATGG	(CTG)7	159	2.8	55
RM1	1	GCGAAAACACAATGCAAAAA	GCGTTGGTTGGACCTGAC	(GA)26	113	29.7	55
RM259	1	TGGAGTTTGAGAGGAGGG	CTTGTTGCATGGTGCCATGT	(CT)17	162	54.2	55
RM237	1	CAAATCCCGACTGCTGTCC	TGGGAAGAGAGCACTACAGC	(CT)18	130	115.2	55
RM212	1	CCACTTTCAGCTACTACCAG	CACCCATTTGTCTCTCATTATG	(CT)24	75	148.7	55
RM226	1	AGCTAAGGTCTGGGAGAAACC	AAGTAGGATGGGGCACAAGCTC	(AT)38	274	154.8	55
RM431	1	TCCTGCGAACTGAAGAGTTG	AGAGCAAAACCCTGGTTCAC	(AG)16	251	178.3	55
RM3874	2	TGGGTGATCTTAGTTTGGCC	AATGTGCCTGCACATGTCAC	(GA)51	206	94.3	55
RM6318	2	TGCTGCTTCTGTCCAGTGAG	GGATCATAACAAGTGCCTCG	(CTT)12	199	96.6	55
RM106	2	CGTCTTCATCATCGTCGCCCCG	GGCCCATCCCGTCGTGGATCTC	(GAA)5	253	123.2	55
RM250	2	GGTTCAAACCAAGCTGATCA	GATGAAGGCCTTCCACGCAG	(CT)17	153	170.1	55
RM213	2	ATCTGTTTGCAGGGGACAAG	AGGTCTAGACGATGTCGTGA	(CT)17	139	186.4	55
RM207	2	CCATTCGTGAGAAGATCTGA	CACCTCATCCTCGTAACGCC	(CT)25	118	191.2	55
RM48	2	TGTCCCACTGCTTTCAAGC	CGAGAATGAGGGACAAATAACC	(GA)17	204	190.2	55
RM535	2	ACTACATACACGGCCCTTGC	CTACGTGGACACCGTCACAC	(AG)11	138	195.7	55
RM166	2	GGTCCTGGGTCAATAATTGGGTTACC	TTGCTGCATGATCCTAAACCGG	(T)12	321	183	61
RM3688	2	GTTGAATCAAGCTGTGCAGC	AGCTAGGCAAAGCATGCATG	(GA)15	111	88.2	50
RM1920	2	CAAACACAGTGTTGACAGAA	GCTATTGACTTATCCGTTCA	(AT)18	127	103.4	55
RM6318	2	TGCTGCTTCTGTCCAGTGAG	GGATCATAACAAGTGCCTCG	(CTT)12	199	96.6	55
RM231	3	CCAGATTATTTCCTGAGGTC	CACTTGCATAGTTCTGCATTG	(CT)16	182	15.7	55
RM251	3	GAATGGCAATGGCGCTAG	ATGCGGTTCAAGATTCGATC	(CT)29	147	79.1	55
RM16	3	CGCTAGGGCAGCATCTAAA	AACACAGCAGGTACGCGC	(TCG)5(GA)16	181	131.5	55
RM55	3	CCGTCGCCGTAGTAGAGAAG	TCCCGGTTATTTTAAGGCG	(GA)17	226	168.2	55
RM1352	3	ACGAGTTGTACTCTGGTTGC	TCTCGGTTTTTATCTTGCTG	(AG)23	219	145.6	55
RM514	3	AGATTGATCTCCCATTCCCC	CACGAGCATATTACTAGTGG	(AC)12	259	216.4	55
RM565	3	AGTAACGAGCATAGCAGGCG	GCAAAGCCTTCAGGAATCAG	(GA)11	172	215.5	55
RM570	3	GTTCTTCAACTCCCAGTGCG	TGACGATGTGGAAGAGCAAG	(AG)15	208	221.1	55
RM85	3	CCAAAGATGAAACCTGGATTG	GCACAAGGTGAGCAGTCC	(TGG)5(TCT)12	45	231	55
RM551	4	AGCCCAGACTAGCATGATTG	GAAGGCGAGAAGGATCACAG	(AG)18	153	8.5	55

RM32	4	AGTCTACGTGGTGTACACGTGG	TGCGGCCTGCCGTTTGTGAG	(TC)3A(CT)9(TC)5	168	4.2	55
RM307	4	GTACTACCGACCTACCGTTCAC	CTGCTATGCATGAACTGCTC	(AT)14(GT)21	174	0	55
RM348	4	CCGCTACTAATAGCAGAGAG	GGAGCTTTGTTCTTGCGAAC	(CAG)7	103	160.8	55
RM122	5	GAGTCGATGTAATGTCATCAGTGC	GAAGGAGGTATCGCTTTGTTGGAC	(GA)7A(GA)2A(GA)11	227	2.2	55
RM574	5	GGCGAATTCTTTGCACTTGG	ACGGTTTGGTAGGGTGTCAC	(GA)11	155	41	55
RM8039	5	CGTACGTACTTATATCTCAT	AAATCTAATGTATCTGAGGT	(TTA)57	260	13.3	55
RM163	5	ATCCATGTGCGCCTTTATGAGGA	CGCTACCTCCTTCACTTACTAGT	(GGAGA)4(GA)11C(GA)20	130	91.4	55
RM31	5	GATCACGATCCACTGGAGCT	AAGTCCATTACTCTCCTCCC	(GA)15	140	134.9	55
RM13	5	TCCAACATGGCAAGAGAGAG	GGTGGCATTCGATTCCAG	(GA)6-(GA)16	141	28.6	55
RM510	6	AACCGGATTAGTTTCTCGCC	TGAGGACGACGAGCAGATTC	(GA)15	121	20.8	55
RM204	6	GTGACTGACTTGGTCATAGGG	GCTAGCCATGCTCTCGTACC	(CT)44	169	25.1	55
RM162	6	GCCAGCAAAACCAGGGATCCGG	CAAGGTCTTGTGCGGCTTGCGG	(AC)20	186	108.3	61
RM125	7	ATCAGCAGCCATGGCAGCGACC	AGGGGATCATGTGCCGAAGGCC	(GCT)8	127	24.8	55
RM214	7	CTGATGATAGAAACCTCTTCTC	AAGAACAGCTGACTTCACAA	(CT)14	108	34.7	55
RM11	7	TCTCCTCTTCCCCCGATC	ATAGCGGGCGAGGCTTAG	(GA)17	91	47	55
RM1132	7	ATCACCTGAGAAACATCCGG	CTCCTCCCACGTCAAGGTC	(AG)12	93	83.3	55
RM248	7	TCCTTGTGAAATCTGGTCCC	GTAGCCTAGCATGGTGCATG	(CT)25	82	144.1	55
RM408	8	CAACGAGCTAACTTCCGTCC	ACTGCTACTTGGGTAGCTGACC	(CT)13	128	0	55
RM3819	8	ACCTCACCTGTGGATCTTGG	CAATCCCCTTCTCTCCTTCC	(GA)20	208	3	55
RM25	8	GGAAAGAATGATCTTTTCATGG	CTACCATCAAAACCAATGTTC	(GA)18	146	52.2	55
RM1384	8	TTAATCCATCCTGTAGCTGG	TCGCTATCAACACTACCTGC	(AG)36	178	11.8	55
RM223	8	GAGTGAGCTTGGGCTGAAAC	GAAGGCAAGTCTTGGCACTG	(CT)25	165	80.5	55
RM284	8	ATCTCTGATACTCCATCCATCC	CCTGTACGTTGATCCGAAGC	(GA)8	107	83.7	55
RM316	9	CTAGTTGGGCATACGATGGC	ACGCTTATATGTTACGTCAAC	(GT)8-(TG)9(TTTG)4(TG)4	192	1.8	55
RM257	9	CAGTTCCGAGCAAGAGTACTC	GGATCGGACGTGGCATATG	(CT)24	163	66.1	55
RM215	9	GAGCAGCAAGAGCAGCAGAGG	CATGCTCGACTTCAGAAGCTTGG	(CT)16	173	99.4	55
RM434	9	GCCTCATCCCTCTAACCCTC	CAAGAAAGATCAGTGCGTGG	(TC)12	152	57.7	55
RM257	9	CAGTTCCGAGCAAGAGTACTC	GGATCGGACGTGGCATATG	(CT)24	147	66.1	55
RM288	9	CCGGTCAGTTCAAGCTCTG	ACGTACGGACGTGACGAC	(GA)7G6(GA)7	125	74.6	55
RM474	10	AAGATGTACGGGTGGCATTC	TATGAGCTGGTGAGCAATGG	(AT)13	213	0	55
RM271	10	TCAGATCTACAATTCCATCC	TCGGTGAGACCTAGAGAGCC	(GA)15	101	59.4	55
RM6100	10	TCCTCTACCAGTACCGCACC	GCTGGATCACAGATCATTGC	(CGA)8	144	53.9	50
RM484	10	TCTCCCTCCTCACCATTGTC	TGCTGCCCTCTCTCTCTCTC	(AT)9	299	97.3	55
RM552	11	CGCAGTTGTGGATTTCAGTG	TGCTCAACGTTTGACTGTCC	(TAT)13	195	40.6	55
RM116	11	TCACGCACAGCGTGCCGTTCTC	CAAGATCAAGCCATGAAAGGAGGG	(CT)9	234	26.5	55

RM287	11	TTCCCTGTTAAGAGAGAAATC	GTGTATTTGGTGAAAGCAAC	(GA)21	118	68.6	55
RM209	11	ATATGAGTTGCTGTCGTGCG	CAACTTGCATCCTCCCCTCC	(CT)18	88	73.9	55
RM206	11	CCCATGCGTTTAACTATTCT	CGTTCCATCGATCCGTATGG	(CT)21	134	102.9	55
RM224	11	ATCGATCGATCTTCACGAGG	TGCTATAAAAGGCATTCGGG	(AAG)8(AG)13	157	120.1	55
RM277	12	CGGTCAAATCATCACCTGAC	CAAGGCTTGCAAGGGAAG	(GA)11	124	57.2	55
RM235	12	AGAAGCTAGGGCTAACGAAC	TCACCTGGTCAGCCTCTTTC	(CT)24	124	101.8	55
RM415	12	CTTCGATCCATCATCCATGG	ATTGCTGTACGCAGTTTCGG	(AT)21	227	0	55
RM19	12	CAAAAACAGAGCAGATGAC	CTCAAGATGGACGCCAAGA	(ATC)10	226	20.9	55

List of markers used for the screening which are linked to reported QTLs

SSR	Chromosome number	Associated QTL	Forward primer	Reverse primer	Repeat motifs
RM 5	1	*yldp1.4*	TGCAACTTCTAGCTGCTCGA	GCATCCGATCTTGATGGG	(GA)14
RM9	1	*yldp1.4*	GGTGCCATTGTCGTCCTC	ACGGCCCTCATCACCTTC	(GA)15GT(GA)2
RM250	2	*yldp2.3, dtm2.7, nsp1.2*	GGTTCAAACCAAGCTGATCA	GATGAAGGCCTTCCACGCAG	(CT)17
RM535	2	*yldp2.3, bm2.2*	ACTACATACACGGCCCTTGC	CTACGTGGACACCGTCACAC	(AG)11
RM434	9	*yldp9.1*	GCCTCATCCCTCTAACCCTC	CAAGAAAGATCAGTGCGTGG	(TC)12
RM166	2	*dtm2.7, nsp1.2, bm2.2*	GGTCCTGGGTCAATAATTGGGTTACC	TTGCTGCATGATCCTAAACCGG	(T)12
RM415	12	*nt12.1*	CTTCGATCCATCATCCATGG	ATTGCTGTACGCAGTTTCGG	(AT)21
RM3688	2	-	GTTGAATCAAGCTGTGCAGC	AGCTAGGCAAAGCATGCATG	(GA)15
RM1920	2	-	CAAACACAGTGTTGACAGAA	GCTATTGACTTATCCGTTCA	(AT)18
RM257	9	*yldp9.1, nfg1.2*	CAGTTCCGAGCAAGAGTACTC	GGATCGGACGTGGCATATG	(CT)24
RM288	9	*nfg1.2*	CCGGTCAGTTCAAGCTCTG	ACGTACGGACGTGACGAC	(GA)7G6(GA)7
RM6318	2	-	TGCTGCTTCTGTCCAGTGAG	GGATCATAACAAGTGCCTCG	(CTT)12
RM19	12	*nt12.1*	CAAAAACAGAGCAGATGAC	CTCAAGATGGACGCCAAGA	(ATC)10
